# Organotypic slice culture model demonstrates inter-neuronal spreading of alpha-synuclein aggregates

**DOI:** 10.1186/s40478-019-0865-5

**Published:** 2019-12-19

**Authors:** Sara Elfarrash, Nanna Møller Jensen, Nelson Ferreira, Cristine Betzer, Jervis Vermal Thevathasan, Robin Diekmann, Mohamed Adel, Nisreen Mansour Omar, Mohamed Z. Boraie, Sabry Gad, Jonas Ries, Deniz Kirik, Sadegh Nabavi, Poul Henning Jensen

**Affiliations:** 1 Danish Research Institute of Translational Neuroscience – DANDRITE, Aarhus University, Aarhus, Denmark; 2 Department of Biomedicine, Aarhus University, Aarhus, Denmark; 3 Department of Medical Physiology, Mansoura University, Mansoura, Egypt; 4 European Molecular Biology Laboratory, Cell Biology and Biophysics Unit, Heidelberg, Germany; 5 Collaboration for joint PhD degree between EMBL and Heidelberg University, Faculty of Biosciences, Heidelberg, Germany; 6 Fakeeh college of Biomedical Sciences, Jeddah, Kingdom of Saudi Arabia; 7 Brain Repair and Imaging in Neural Systems (BRAINS) Unit, Department of Experimental Medical Science, Lund University, Lund, Sweden; 8 Department of Molecular Biology and Genetics, Aarhus University, Aarhus, Denmark

**Keywords:** Alpha-synuclein, Prion-like spreading, Serine-129 phosphorylation, Organotypic slices

## Abstract

Here we describe the use of an organotypic hippocampal slice model for studying α-synuclein aggregation and inter-neuronal spreading initiated by microinjection of pre-formed α-synuclein fibrils (PFFs). PFF injection at dentate gyrus (DG) templates the formation of endogenous α-synuclein aggregates in axons and cell bodies of this region that spread to CA3 and CA1 regions. Aggregates are insoluble and phosphorylated at serine-129, recapitulating Lewy pathology features found in Parkinson’s disease and other synucleinopathies. The model was found to favor anterograde spreading of the aggregates. Furthermore, it allowed development of slices expressing only serine-129 phosphorylation-deficient human α-synuclein (S129G) using an adeno-associated viral (AAV) vector in α-synuclein knockout slices. The processes of aggregation and spreading of α-synuclein were thereby shown to be independent of phosphorylation at serine-129. We provide methods and highlight crucial steps for PFF microinjection and characterization of aggregate formation and spreading. Slices derived from genetically engineered mice or manipulated using viral vectors allow testing of hypotheses on mechanisms involved in the formation of α-synuclein aggregates and their prion-like spreading.

## Introduction

Parkinson’s disease (PD) is characterized by the appearance of abnormal proteinaceous inclusions, Lewy bodies, whose development progresses through the nervous system. The pathology has been hypothesized to originate in the gut and olfactory bulb and spread from there through vulnerable neuronal populations in the brain [1, 2]. Alpha-synuclein (α-syn) is a 14 kDa protein extensively expressed in the mammalian brain with several recent reports providing evidence for its expression in other central and peripheral tissues. The exact function of α-syn still needs further investigation, but the available data suggest a role of presynaptically expressed α-syn in synaptic transmission, particularly in trafficking and release of vesicles, based on the close association of α-syn and the SNARE complex proteins [3, 4]. The presence of α-syn in the nucleus has also led to the suggestion of a role in transcription either through indirect interaction with DNA including modulation of histone modification state [5, 6] or through direct binding to the DNA [7–9].

The interest in α-syn in relation to PD stems from autopsy studies, reporting α-syn as a main component of the Lewy pathology in the brains of PD patients [10]. The progressive spreading of Lewy pathology through different brain regions of PD patients has suggested an intercellular transfer of seeding-competent α-syn aggregates from affected neurons to healthy ones. Upon uptake, aggregation of native α-syn into toxic and seeding-competent aggregates is templated in a process that can perpetuate throughout the nervous system [1, 11, 12]. This hypothesis is supported by several in vivo experiments where injection of seeds, consisting of pre-formed α-syn fibrils or α-syn aggregate-containing brain extracts, induces spreading of α-syn pathology [11, 13–15]. It is further substantiated by cell-based models that allow mechanistic studies of seed uptake, aggregation process, intercellular spreading of newly formed aggregates and drug screening [16–18]. However, the complexity of in vivo models on one hand and the simplicity of cell-based models on the other hand often hamper the study of mechanisms operating in the brain. For these studies, the use of ex vivo models like organotypic slices could be a promising tool to establish and use in the field.

Here, we introduce a novel ex vivo model using organotypic hippocampal slices that allow the study of inter-neuronal spreading of α-syn aggregate pathology on a shorter time scale than in previously known in vivo animal models. In addition to this, the model provides the ease of manipulation known from cell-based models by combining organotypic mouse hippocampal cultures with region-specific microinjection of pre-formed α-syn fibrils (PFFs). Here, we demonstrate and highlight the essential steps of slice preparation, PFF microinjection and characterization of the PFF-induced aggregation. Using the slice model, we demonstrate spreading of α-syn aggregation in the anterograde direction from DG to CA3 and CA1 pyramidal neurons but not in the opposite, retrograde manner. As an example of an application of the model, the role of phosphorylation at S129 (pS129) is investigated. A growing body of literature has examined the role of pS129 post-translational modification in regulation of the aggregation process, with contradicting results reporting both increases and decreases of insoluble aggregates. Likewise, in vitro studies have reported both increases and inhibition of α-syn aggregation into insoluble structures following serine-129 phosphorylation [19–22]. Using organotypic hippocampal slice cultures (OHSCs) expressing only S129G-mutated α-syn, we show that pS129 is not required neither for aggregation nor spreading of the α-syn pathology, even though this post-translational modification is generally accepted as a marker of Lewy pathology.

The swiftness of the templated α-syn aggregate development and spreading in the model combined with its flexibility with respect to using tissue from genetically modified mouse strains and viral vector technology, opens up for new ways of investigating molecular mechanisms operating in the spreading of α-syn pathology in the brain. Thus, the slice model could make up for the current lack of models for studying the impact of templated α-syn aggregation on neuronal functionality and connectivity, e.g. through electrophysiology and live-cell imaging.

## Materials and methods

### Preparation of organotypic hippocampal slice cultures

Organotypic hippocampal slices were made from 5 to 7-day-old post-natal pups from C57BL/6 (wild type, WT), SNCA^−/−^ α-syn knockout (α-syn KO; C57BL/6 N-Sncatm1Mjff/J from Jackson lab), or mThy-1-human α-syn-expressing mice (Tg(Thy1-SNCA)61Ema) [23] according to Stoppini et al. [24]. Low Na cerebrospinal fluid (CSF; 1 mM CaCl_2_, 10 mM D-glucose, 4 mM KCl, 5 mM MgCl_2_, 26 mM NaHCO_3_, 234 mM sucrose and 0.1% phenol red solution) was carbogenated on ice until the color changed to orange and some ice lumps had formed. 15 mL of low Na CSF was prepared for each brain in a 50 mL Falcon tube. After decapitation of the pups, the extracted brain was gently removed and kept in low Na CSF for 1 min, before pouring the brain and low Na CSF into a petri dish for hippocampus dissection under microscopic guidance (SZX-ZB7 Stereomicroscope, Olympus × 1 objective). Slices of 400 μm thickness were made using a tissue chopper (Stoelting, #51425) and moved to a dish with pre-heated culture medium (MEM Eagle medium 78.8% (Gibco #11095), 20% heat-inactivated horse serum (Gibco, #16050–122), 1 mM L-glutamine, 1 mM CaCl_2_, 2 mM MgSO_4_, 170 nM insulin, 0.0012% ascorbic acid, 12.9 mM D-glucose, 5.2 mM NaHCO_3_, 300 mM Hepes (Sigma, #H3375), pH = 7.28, osmolality adjusted to 317–322). Slices with intact DG and CA regions were selected under the microscope and moved to air-fluid interface-style Millicell culture inserts (Millipore, #PICM0RG50) in 6-well culture plates (ThermoFisherScientific) with 800 μL of sterile medium added below the insert [24]. The medium was changed completely thrice weekly. All steps of the procedure after decapitation and brain extraction were performed in a laminar flow tissue culture hood using sterile equipment and aseptic technique.

### Preparation of WT and S129A α-syn pre-formed fibrils

Recombinant human α-syn with residue serine-129 mutated to an alanine or WT human α-syn was expressed in *E. coli* and purified as described for wild type α-syn [25]. To make pre-formed WT or S129A-α-syn fibrils, monomeric WT or S129A α-syn (5 mg/mL in sterile phosphate-buffered saline (PBS), pH 7.4 (Gibco)) was incubated in Eppendorf tubes for 48 h at 37 °C on a Thermomixer (Eppendorf) at 1050 rpm. To validate sufficient aggregation, 50 μL of the incubated solution was centrifuged at 25,000×*g* for 20 min to separate supernatant and pellet. Pellet was resuspended in 50 μL PBS, and 50 μL 2 x SDS-loading buffer (20 mM Tris, pH 6.8, 2 mM EDTA, 80 mM DTT, 2% SDS, 20% sucrose) was added to both pellet and supernatant, which were heated to 96 °C for 15 min. Equal volumes of supernatants and pellets were subjected to sodium dodecyl sulfate polyacrylamide gel electrophoresis (SDS-PAGE) analysis on 8–16% Bis-Tris gels (Genscript) and subsequently stained with Coomassie blue R-250 (Additional file 1: Figure S1a,i & b,i). Approximately 75% of the protein was routinely recovered in the insoluble pellet fraction. To ascertain the development of amyloid-type aggregates in the insoluble fractions, K114 fluorometry was conducted [26]. Equal volumes of aggregated and monomer α-syn were mixed with (trans,trans)-1-bromo-2,5-bis-(4-hydroxy)styrylbenzene (K114) (50 μM) in 100 mM glycine, pH 8.5, after which fluorescence (λ_ex_ = 380 nm, λ_em_ = 550 nm, cutoff = 530 nm) was measured with a Perkin Elmer EnSpire 2300 Multilabel Reader (Additional file 1: Figure S1a,ii & b,ii).

When proper aggregation had been assured, the insoluble WT or S129A α-syn was isolated by centrifugation in Eppendorf tubes. After discarding the supernatant, the pellet (containing the PFFs) was diluted to 2 mg/mL in sterile PBS, pH 7.4 (Gibco) and subjected to ultrasound breakage for 20 min using a sonicator (Branson 250, settings: 30% Duty Cycle, Output Control 3) equipped with a water jacket cooling system to avoid sample heating during sonication. The size distribution profile of the PFFs was measured by dynamic light scattering (DLS) using a Wyatt DynaPro NanoStar instrument at 25 °C. Data were processed using the Dynamics 7.5.0.17 software package with the solvent (PBS) background signal subtracted from each sample. The PFFs sample comprised homogeneous, monodispersed populations with a hydrodynamic radius of 44 nm (S129A PFFs) or 38 nm (WT PFFs) (Additional file 1: Figure S1a,iii & b,iii). The PFFs were dispensed into sterile Eppendorf tubes in (75 μL) aliquots with a concentration of 2 mg/mL, snap frozen and stored at − 80 °C. Protein concentration of the aliquots was validated using the bicinchoninic acid assay (BCA) kit (Sigma).

### Microinjection of organotypic slices

To facilitate injection, each insert was moved from the 6-well plate to a sterile cell culture dish, 35 × 10 mm (SARSTEDT #83.3900.500). Slices were microinjected in the DG after 7 days in culture, except when otherwise mentioned. Light microscopy was used to identify the DG by the characteristic horseshoe arrangement of the nuclei of granule cells.

Immediately before injection, an aliquot of WT or S129A PFFs was thawed at room temperature (RT) and sonicated for 30 s using the above-mentioned settings (Branson, Sonifier 250). After sonication, PFFs were kept at RT during the injection process. Microinjection pipettes (item #1B200F-4 (with Filament), WPI) were pulled using a micropipette puller (P-1000, Sutter Instrument, settings: heat 590, pull 80, velocity 90, time 125, pressure 400, ramp 523). For microinjection, a Pulse Pal v2 (#1102) was used (settings: phase 1 voltage 5 V, phase 1 duration 0.01 s, pulse interval 0.5 s).

Injections were performed in a laminar flow hood equipped with a microscope to ensure aseptic conditions. The pipette was loaded using Eppendorf microloader pipette tips (ThermoFisher), inserted into the holder, and the tip was cut with a pair of fine scissors under visual guidance. Pressure pulse was applied to test whether the PFF suspension was expelled from the tip, appearing as a small droplet at the tip of the needle. Correct injection was confirmed by temporary lifting of the surface of the tissue at the injection site. After injection, the needle was left in place for 20 s and then slowly removed. The volume injected in the slices was estimated by counting the number of shots with the adjusted Pulse Pal set up (10–12 shots/slice, 10 nL/shot, 0.1 μg/slice). The final volume was injected at the DG at two to three injection sites depending on the slice thickness at the site of needle insertion. It is important to pay attention to tissue architecture under microscopic guidance during the injection procedure to avoid injection of a large volume at a single site, which will cause rupture of the tissue and release of the PFFs to the surface of the OHSC. The session lasted approximately 6 to 8 min for an insert holding four slices. The final volume injected in each slice was about 0.1 μL of either WT PFFs (1 mg/mL), S129A PFFs (1 mg/mL), monomeric α-syn (1 mg/mL), or PBS. After injecting all slices on a culture insert, the medium was replaced with fresh, pre-heated medium.

### Adeno-associated virus-mediated expression of WT and S129G α-syn in slices from α-syn KO mice

Pseudotyped rAAV2/6 WT human α-syn and rAAV2/6 S129G human α-syn vectors were produced using a co-transfection method with an rAAV transfer plasmid containing the gene of interest placed between two AAV2 inverted terminal repeats and a helper plasmid (pDP6) coding for necessary elements for production and packaging of the capsid particles. The gene of interest was expressed under the control of a human synapsin-1 promoter. Vectors were purified by iodixanol step gradients and ion exchange chromatography, as described in detail elsewhere [27]. The final titers used in the experiments were 3.5 × 10^13^ genome copies/mL. All titers were determined by quantitative PCR using TaqMan probes targeting the ITR sequences. After 3 days in culture, AAV-human-WT-α-syn and AAV-human-S129G-α-syn vectors were injected in slices made from P5 α-syn KO pups. The slices were either injected with the virus at the three regions DG, CA3, and CA1 or only at DG and CA1. This procedure expresses α-syn in either all three synaptically connected regions or leaves the interconnecting CA3 region without α-syn expression. Three days after AAV injection, S129A PFFs were injected at DG as described above.

### Immunohistochemistry

Organotypic slices were fixed using 4% PFA in PBS (2.8 mM NaH_2_PO_4_H_2_O, 7.2 mM Na_2_HPO_4_.2H_2_O, 123 mM NaCl, pH-adjusted to 7.2) and processed for immunohistochemistry according to Gogolla et al., with slight modifications [28]. Briefly, after fixation, slices were permeabilized in 0.5% Triton X-100 for 6 h at room temperature (RT) or overnight at 4 °C with slight shaking. Slices were then incubated with blocking buffer (10% bovine serum albumin (BSA)/PBS) for 3 h at RT. Primary antibody was prepared in 5% BSA/PBS and incubated with slices overnight at 4 °C with gentle shaking. Antibodies used were α-syn aggregate-specific antibody MJF-14 (rabbit mAb MJFR-14-6-4-2 #ab209538, Abcam, 1:25,000), two phospho-serine-129-specific antibodies, 11A5 [29] (mouse monoclonal 11A5 kindly provided by Imago Pharmaceuticals, 1:25,000) and D1R1R (rabbit mAb #23706, Cell Signaling, 1:1000), neurofilament light chain (NF-L; mouse mAb #2835, Cell Signaling, 1:500), NeuN (mouse mAb clone A60 #MAB377, Millipore, 1:200), and anti-alpha-synuclein antibody MJFR1 (rabbit mAb #ab138501, Abcam, 1:5000). The slices were washed three times in a TBS washing buffer (NaCl 150 mM, Tris 20 mM, 0.3% Triton X-100) for 30 min/wash with gentle shaking. After the final wash, slices were incubated with the appropriate Alexa Fluor dye (488 and 568) labelled secondary antibodies (Invitrogen, 1:2000) and 4′,6-diamidino-2-phenylindole (DAPI) (TH.GEYER, 5 mg/mL, 1:1000) in 5% BSA/PBS for 3 h at RT with gentle agitation and shielded from light. The slices were washed three times as above and mounted on glass slides using DAKO fluorescence mounting medium (DAKO, S3023). The edges of the coverslips were sealed with nail polish.

The staining can be done either by i) directly adding reagents to the inserts (1 mL above and 1 mL below the inserts) or ii) excising the slices from the inserts with their culture membrane below using a scalpel and then incubating them with antibodies in 96-well plates to save reagents. For the washing steps, 24-well plates were used to ensure proper rinsing.

### Preparation of organotypic hippocampal slice cultures for super-resolution dSTORM imaging

Before cryosectioning, 4% PFA-fixed slices were kept in 30% sucrose at 4 °C overnight for cryoprotection. The slices were cut out of the membrane insert with a piece of membrane below and mounted on the cryostat stage using Tissue-Tek® O.C.T. Compound (Sakura), with the slice surface facing downwards. Once the OCT had solidified, the membrane was carefully removed and an extra layer of OCT was added on top of the slice and left to freeze completely. The OHSCs were then cut on a cryostat (Leica, #CM1900) at a thickness of 10 μm and a temperature of − 20 °C. The sections were collected on Superfrost Plus Adhesion Microscope Slides (ThermoFisher) and stored at − 20 °C. Immunostaining was processed on the slides as previously described for the whole mount using an antibody against pS129 (D1R1R, rabbit mAb #23706S, Cell Signaling, 1:1000). During incubations, slides were kept in a humidity chamber with a hydrophobic pen barrier drawn around the tissue to prevent it from drying out. After the final washing step, the slides were mounted using glycerol gelatin aqueous slide mounting medium (Sigma, #GG1).

#### Tissue preparation for super-resolution dSTORM imaging

To facilitate dSTORM imaging, conventional dSTORM imaging buffer (50 mM Tris/HCl pH 8, 10 mM NaCl, 10% (w/v) D-Glucose, 500 μg/mL glucose oxidase, 40 μg/mL glucose catalase and 35 mM MEA in H_2_O) was added to the gelatin-embedded slice. The rectangular coverslip was carefully removed by immersing the microscope slide into a beaker of PBS pre-warmed to 60 °C. Imaging buffer (100 μL) was added to the gelatin layer, and a new rectangular coverslip was then placed over the gelatin layer. The coverslip edges were then sealed using a two-component silicone glue. Once the glue had set, the coverslip and microscope slide sandwich was mounted on a microscope stage for imaging.

#### Microscope setup for dSTORM acquisition

dSTORM image acquisition was performed on a custom-built inverted microscope. Laser light at 405 nm and 640 nm wavelengths was emitted from a laser box equipped with a single-mode fiber (iChrome MLE, Toptica), collimated using an achromatic lens (f = 30 mm, Thorlabs), relayed by a 4f microscope of two lenses (each f = 250 mm, Thorlabs), spatially filtered for background reduction by an iris conjugate to the image plane (SM1D12D, Thorlabs), and focused onto the back-focal plane of a water-immersion objective lens (60x/NA1.2, Olympus). Mounting the fiber on a one-axis stage (SLC2445me-4, Smaract) allowed for image acquisition in HILO (highly inclined and laminated optical sheet) mode [30]. Fluorescence excitation and emission was separated using a multi-line dichroic beam splitter (zt405/488/561/640rpc, Chroma). Fluorescence emission was additionally filtered by a band pass filter (676/37 BrightLine HC, AHF) and directly focused onto an sCMOS camera (Orca Flash 4.0, Hamamatsu) via a tube lens (f = 180 mm, MVX-TLU, Olympus) resulting in projected pixel widths of 108 nm. Raw dSTORM data were recorded at a frame rate of 40 Hz and 25 ms exposure time. The lasers were triggered using a focal plane array (Mojo, Embedded Micro) controlled by a custom-written Micro Manager 1.4.22 plugin [31]. This kept the number of activated emitters per frame approximately constant to a preset value via modulation of the pulse length of the 405 nm laser. The intensity of the fluorescence excitation laser at 640 nm was about 10 kW/cm^2^.

### Immunoblotting analysis of brain slice extracts

Slices were collected by cutting out a small square of membrane for each slice to ensure that all tissue was collected. At each time point, eight slices were collected. After 14 days in culture (7 days post injection of PFFs), around 30 μg of protein was extracted per slice.

After washing twice in Hank’s buffer (5.37 mM KCl, 0.44 mM KH_4_PO_4_, 0.44 mM Na_2_HPO_4_, 136.9 mM NaCl), the tissue was homogenized with a tissue homogenizer (VWR #4320202) in ice-cold radioimmunoprecipitation assay (RIPA) buffer (50 mM Tris (pH 7.4), 150 mM NaCl, 1% Triton X-100, 2 mM EDTA, 0.5% sodium deoxycholate, 0.1% SDS) supplemented with protease inhibitor cocktail (cOmplete, Roche) and phosphatase inhibitors (25 mM β-glycerolphosphate, 5 mM NaF, 1 mM Na_3_VO_4_, 10 mM Na-pyrophospate). After homogenization, samples were sonicated in Eppendorf tubes (Branson, Sonifier 250, settings: 30% Duty Cycle, Output Control 3, 40 shots) before centrifugation at 25,000×*g* for 25 min at 4 °C. Supernatants were collected as the RIPA-soluble fraction. The pellets were washed twice by resuspension in RIPA buffer and centrifuged to remove remaining soluble material from the insoluble pellets. The pellets, constituting the RIPA-insoluble aggregate fraction, were then dissolved in SDS-urea buffer (4% SDS, 50 mM Tris, 7 M urea, 40% glycerol and bromophenol blue, 2.5 mM DTE) overnight at RT. Protein concentration was measured using the BCA assay (Sigma).

The RIPA-soluble fraction lysates were supplemented with SDS-PAGE loading buffer (50 mM Tris pH 6.8, 4% SDS, 2.5 mM DTE, 40% glycerol, bromophenol blue), whereas the RIPA-insoluble fraction was ready for loading on the gel. Samples were heated to 95 °C for 5 min and resolved on 8–16% Bis-Tris gels (Genscript) before blotting onto PVDF membranes using the Iblot2 Dry blotting system (ThermoFisher). Membranes were fixed in 4% PFA for 30 min and blots probed for α-syn were boiled in PBS for 10 min to improve immunodetection of α-syn [32]. Membranes were blocked for 1 h at RT in blocking buffer (5% skimmed milk powder, 20 mM Tris base, 150 mM NaCl, 0.05% Tween 20, containing phosphatase inhibitors) supplemented with 0.02% NaN_3._ Primary and secondary antibodies were diluted in blocking buffer. Incubation with primary antibodies was done overnight at 4 °C and with secondary antibodies (DAKO, #P0217, #P0260) for 1.5 h at RT with washing in TBS-Tween three times 15 min after each incubation. Bound antibodies were visualized using enhanced chemiluminescence in a Fuji LAS-3000 Intelligent Dark Box (Fujifilm, Japan). To reprobe filters, they were stripped for bound antibodies using Restore Western Blot Stripping Buffer (ThermoScientific, #21059) according to the manufacturer’s recommendation. Membranes were then processed with blocking and antibody detection as mentioned above. Antibodies used were the following: rabbit polyclonal anti-α-syn (ASY-1 1:1000) [33], rabbit mAb anti-α-syn antibody (MJFR1 #ab138501, Abcam, 1:1000), mouse mAb pS129-α-syn (11A5, kindly provided by Imago Pharmaceuticals, 1:2000), mouse mAb anti-β-Tubulin III (TUJ1 #T8578, Sigma, 1:5000), rabbit mAb mouse-specific α-syn (D37A6 XP Rabbit #4179, Cell Signaling, 1:1000), mouse mAb anti-α-syn Syn-1 (Clone 42 #610787, BD Transduction Laboratories, 1:1000). PageRuler pre-stained protein ladder 10–180 kDa (ThermoFisher, #26616) was used as the molecular size marker.

### Quantification

Quantification of Western blots was done using ImageJ (National Institutes of Health) after first assuring that the bands were not saturated. For quantification of immunostainings, four pictures covering the whole organotypic slice were taken using the × 10 objective, and a threshold was set where only the aggregate-specific signals were visible. The same threshold was applied to all images in an experiment. For analysis, the mean fluorescence intensity (MFI) of the selected aggregate signals was quantified using ImageJ (National Institutes of Health) software. Signals were normalized to the total surface area of each slice detected using the DAPI staining.

### Statistical data and analysis

Statistical analysis was performed using unpaired Student’s T-test for comparison of two groups. Data are presented as means ± standard deviation (SD) *p < 0.05, **p < 0.01, ***p < 0.001. For dSTORM data analysis, all data analysis and image reconstructions were performed with custom software written in MATLAB, which is available as open source (github.com/jries/SMAP). Single molecule events were localized using a Gaussian fitter. Reconstructed images were rendered after filtering the localization table based on localization precision and point spread function width.

## Results

### Progressive accumulation of mouse α-syn in OHSCs and efficient C-terminal truncation of injected α-syn PFFs

**Fig. 1:**
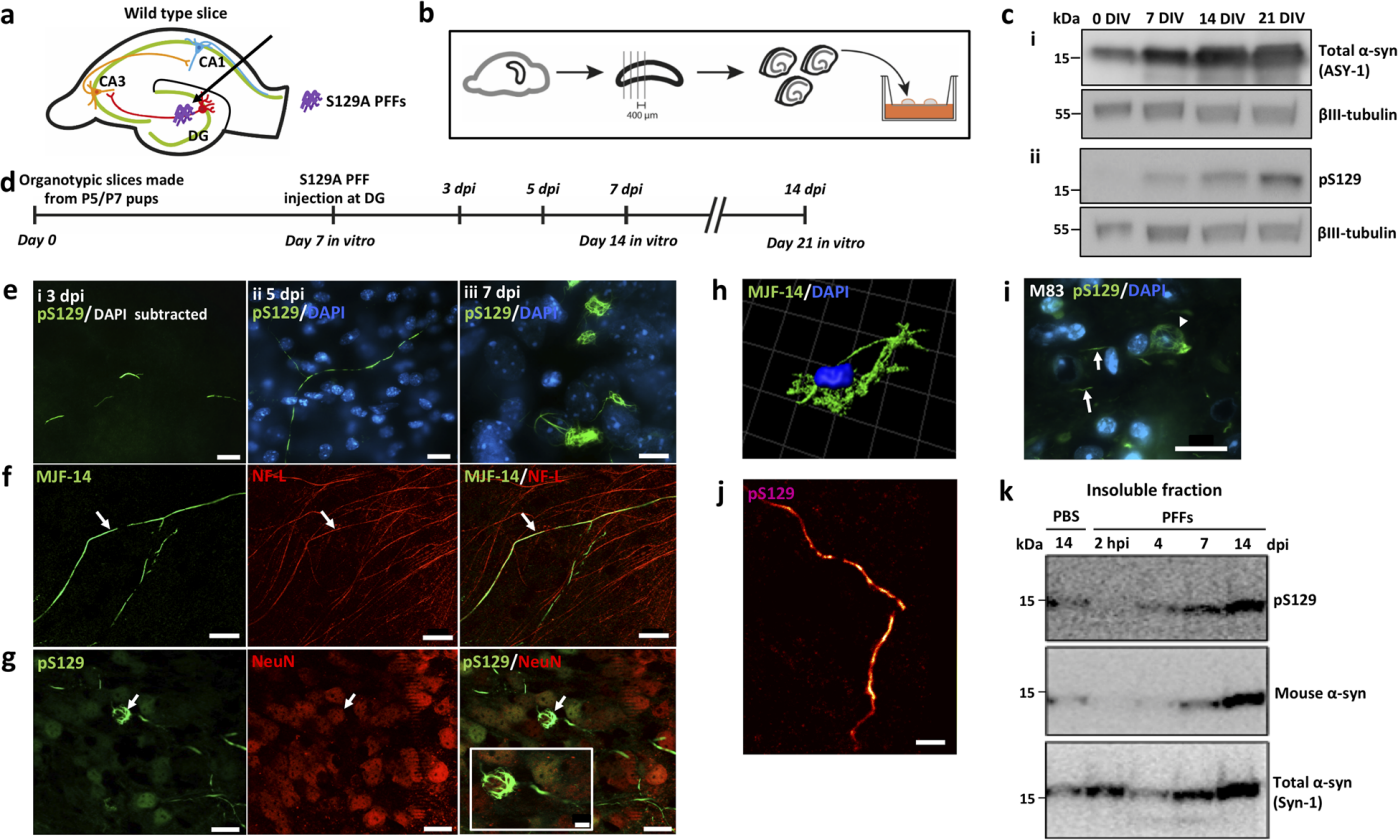
Organotypic mouse hippocampal slice cultures as a model to study seeded α-syn aggregation in the region between DG and CA3. **a** Diagram showing the synaptic connections of granule cells of DG (where S129A PFFs were injected) to pyramidal neurons in CA3 that subsequently connect to the pyramidal neurons of the CA1 region. **b** OHSCs from mouse pups were cultivated on an air-liquid interface. **c** Progressive accumulation of total (**i**) and pS129-α-syn (**ii**, 11A5) in cultures from wild type mouse pups after 0, 7, 14, and 21 DIV analyzed by immunoblotting. **d** Experimental flow showing time of PFF microinjection at 7 DIV and tissue collection for analysis at 3, 5, 7, and 14 dpi. **e** pS129-positive α-syn structures (D1R1R) imaged at DG, following PFF injection at DG. Aggregates are first recognizable at 3 dpi as short serpentine aggregates (**i**) that coalesce into longer aggregates by 5 dpi (**ii**) and at 7 dpi occur as fibrillar aggregates around neuronal nuclei (**iii**). Scale bars: 20 μm. **f** MJF-14-positive serpentine aggregates co-localize with the axonal marker neurofilament light chain (NF-L). Scale bar: 20 μm. **g** pS129-positive cell body inclusions (D1R1R) are located in NeuN-positive neurons. Scale bar: 20 μm, inset: 5 μm. **h** Thread-like cell body inclusion detected by MJF-14 and reconstructed in 3D by IMARIS software. **i** Cell body pS129 α-syn pathology (D1R1R) in the hindbrain of end-stage h-A53T-α-syn transgenic mice (M83) resembles inclusions in the slice model (panels **e,iii** & **g**). **j** dSTORM image reconstruction of pS129-positive axonal processes (D1R1R) within the OHSC. Scale bar: 1 μm. **k** Progressive accumulation of insoluble pS129-positive mouse α-syn (11A5) in PFF-injected slices. Western blots in **c** & **k** are representative of 2–3 separate experiments. Images in **e** are examples from 2 to 6 individual experiments with 9–17 slices in total. Images in **f** & **g** are representative of 4–5 experiments/15–16 slices in total

To study α-syn aggregate pathology and its spreading in brain tissue, we developed a new ex vivo model based on the organotypic mouse hippocampal slice culture method. The slices were prepared from 5 to 7-day-old postnatal pups of wild type BL6 mice, cultivated on a membrane by the air-fluid interphase method [24] (Fig. 1b). This preparation has been extensively used for electrophysiological studies because it exhibits well-characterized synaptic connectivity between granule neurons in DG and CA3 pyramidal neurons that subsequently form synapses on CA1 pyramidal neurons [34] (Fig. 1a).

Because endogenous expression of α-syn is a prerequisite for templating α-syn aggregates and subsequent inter-neuronal spreading, we first determined the development of α-syn expression in wild type brain slices throughout a 21-day culture period. The cultivated slices exhibited a progressive increase of total α-syn mirroring the postnatal expression in mice [35] (Fig. 1c). The phosphorylated pS129-form became detectable after 7 days, after which its level also increased (Fig. 1c). Based on these data combined with the observation that the slices get thinner during culture time, which makes the process of proper injection more challenging at late time points, we chose to initiate the process of aggregation by PFF microinjection in slices cultured for 7 days in vitro (DIV).

As pathological α-syn aggregates are heavily phosphorylated at S129, we used PFFs composed of S129A-mutant α-syn that cannot be phosphorylated at this site. This allowed unambiguous detection of endogenous aggregates by pS129-specific antibodies that do not bind the injected S129A seeds.

The fate of the exogenous S129A PFFs injected into the brain slices was investigated using tissue from α-syn KO pups allowing us to focus on the injected material. The tissue was analyzed at 2 h, 3 days, and 7 days post injection (dpi). To facilitate quantitative immunoblotting, PFFs injected into slices were depolymerized in 7 M urea/4% SDS loading buffer, which allowed their quantification as a monomeric band. Using the MJFR1 antibody that binds to a C-terminal epitope (118–123) [36], we observed a 30% reduction in PFFs after 3 days with no detectable protein remaining after 7 days. However, using the Syn-1 antibody that detects an epitope corresponding to amino acids 91 to 99 [36], we found that the injected PFFs remained in the tissue as a C-terminally truncated species for more than 7 days (Additional file 1: Figure S1c). The Syn-1 antibody did not work well for IHC analysis of the PFF-injected α-syn KO slices to localize the truncated species at 5 and 7 days post injection. Staining with MJFR1 revealed that the injected material was confined to a small area close to the injection site in the DG regions 2 h post injection and disappeared after 7 days as expected from the C-terminal truncation (Additional file 1: Figure S1d).

### Injection of S129A PFFs templates α-syn aggregation in OHSCs

When S129A PFFs were microinjected into the DG of OHSCs from wild type pups, pS129-positive aggregates of mouse α-syn became detectable 3 dpi. It started as short, serpentine-like inclusions with a diameter of about 0.06 μm (Fig. 1e,i). The pS129-positive structures were also positive for the aggregate-specific MJF-14 antibody. By 5 dpi, these structures appeared to coalesce into longer serpentine structures, while at 7 dpi fibrillar aggregates were detected in the cell body around the nucleus (Fig. 1e,ii & iii). The aggregates located in the cell bodies resembled those observed by IHC in the hypothalamic region of end-stage hA53T α-syn transgenic mice (Fig. 1i). In order to justify the use of S129A-mutated PFFs for the study of seeded aggregation, we also injected WT α-syn PFFs in OHSCs to verify that the mutation did not induce noticeable differences in the appearance or timing of endogenous aggregation. Biochemically, the WT PFFs were similar to the S129-mutated ones, regarding insolubility, amyloid structure and size (Additional file 1: Figure S1b). The pattern of aggregation at the DG was comparable between the two types of PFFs, with axonal aggregates manifesting as small structures at 3 dpi, increasing size at 5 dpi and cell body aggregates appearing at 7 dpi (Additional file 2: Figure S2a).

The pS129-positive aggregates in the slices were located in axons and neuronal cell bodies as evidenced by their co-localization with the subcellular markers neurofilament light chain and NeuN (Fig. 1f, g). Upon extraction and western blotting of PFF-injected slices, pS129-positive insoluble α-syn species became detectable after 7 days and increased at 14 days. These results demonstrate that endogenous α-syn is converted into insoluble species phosphorylated at serine-129 upon seeding with PFFs (Fig. 1k). Super-resolution microscopy revealed that the axonal pS129-positive inclusions consisted of smaller structures of more intense immunoreactivity suggestive of discrete α-syn inclusions filling or being moved within the axon (Fig. 1j).

**Fig. 2:**
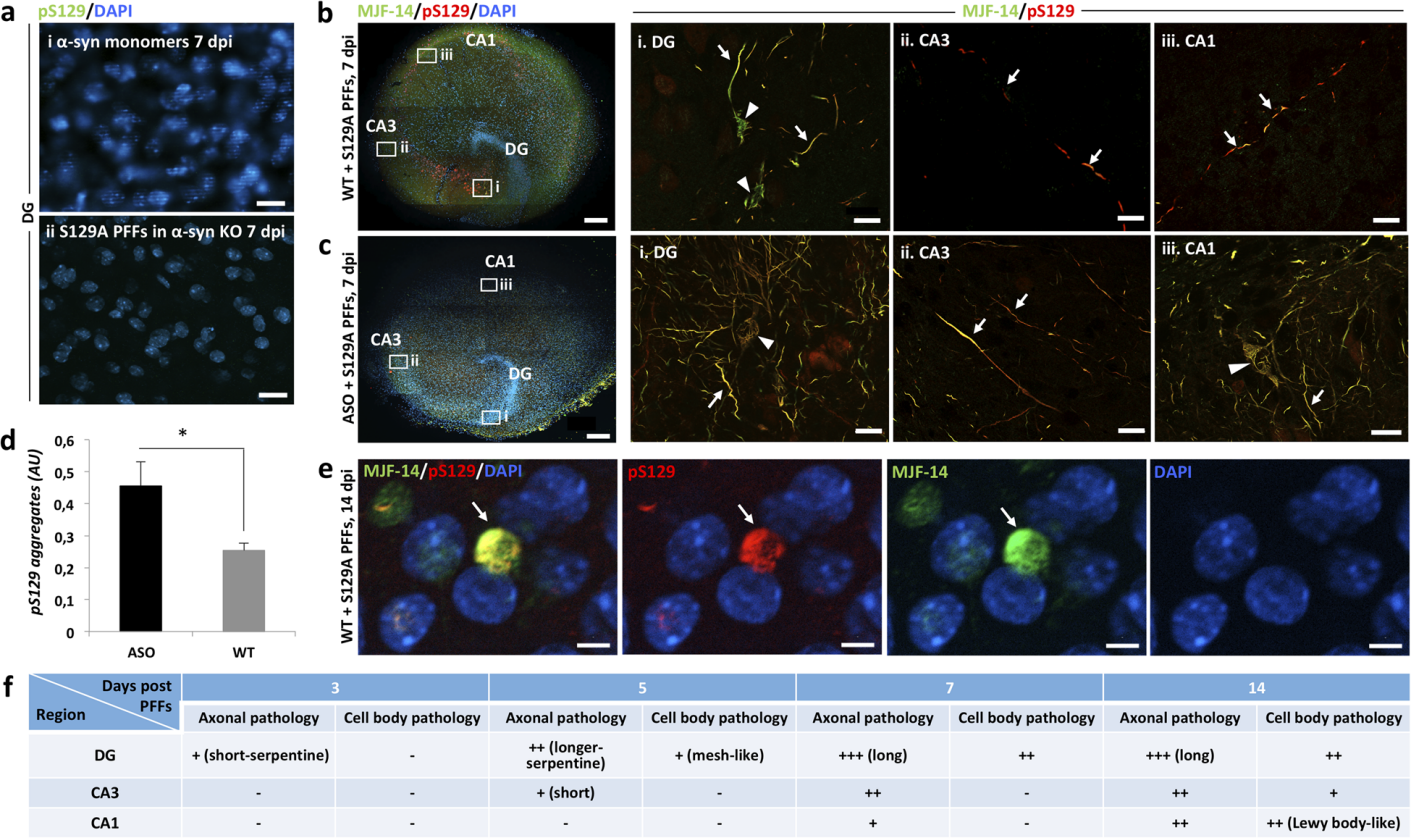
Trans-synaptic spreading of α-syn aggregate pathology from DG via CA3 to the CA1 region depends on α-syn expression levels. **a** No aggregation is induced by injection of (**i**) monomeric α-syn in WT slices or (**ii**) S129A PFFs in α-syn KO slices. Scale bars: 20 μm. **b** Composite image of immunostaining for aggregated (MJF-14, green) and pS129-α-syn (11A5, red) 7 dpi in WT OHSCs, scale bar: 200 μm. Areas from DG, CA3, and CA1 regions indicated are magnified in panels **i**, **ii**, and **iii**. Scale bars: 20 μm. Axonal aggregates (arrows) are present in all three regions, while cell body inclusions (arrowheads) are present only in DG at 7 dpi. **c** Composite image of immunostaining with MJF-14 and pS129 for aggregates 7 dpi in ASO OHSCs. Scale bar: 200 μm. **i**, **ii** Extensive MJF-14- and pS129-positive aggregation and (**iii**) faster progression with development of cell body inclusions in the CA1 region. Scale bars: 20 μm. **d** Quantification of pS129-α-syn aggregate fluorescence signals in total slices from PFF-injected WT and ASO slices. Bars represent mean ± SD, *n* = 3. Unpaired Student’s T-test, *p*-value = 0.019. **e** Immunostaining with pS129 (11A5) and MJF-14 at CA1 region of WT slices 14 dpi of PFFs show more compacted, spherical cytoplasmic inclusions, resembling Lewy bodies. Scale bar: 5 μm. **f** Schematic presentation of progressive development of aggregation; from short into longer serpentine, axonal inclusions in DG regions, which spread to CA3 and CA1 regions. Cell body inclusions appear at later stages when axonal pathology is already established in the region. Images in **a** are illustrative of 2–3 individual experiments with 10–12 slices in total. Images in **b** are representative of 17 slices/6 experiments, while images in **c** represent 3 slices/1 experiment. For quantification in **d**, 3 slices were included per group

Injection of monomeric α-syn into WT slices or injection of S129A PFFs into slices made from α-syn KO pups induced no aggregation at 7 dpi (Fig. 2a). This shows that the aggregation process is dependent on both pre-formed fibrillar seeds and the presence of endogenous α-syn.

### Application 1: demonstrating that S129A PFF-templated α-syn aggregation spreads by inter-neuronal processes from the DG region via CA3 to the CA1 region

Seeded aggregation of endogenous α-syn in the DG region was observed at 3 dpi, while the first inter-neuronal spreading from DG to the CA3 and CA1 regions appeared as axonal inclusions after 5 to 7 days (Fig. 2b). Regarding the timing of seeding away from the injection site, WT α-syn PFFs displayed similar characteristics (Additional file 2: Figure S2b). Neuronal cell body inclusions became detectable in the CA1 region at 14 dpi (Additional file 3: Figure S3), characterized by a compacted, spherical appearance in contrast to the cell body inclusions of the DG. These structures displayed immunoreactivity towards both pS129 and MJF-14 antibodies, suggesting the development of Lewy body-like inclusions at this stage in the CA1 region of the cultures (Fig. 2e & Additional file 4: Figure S4). Both seeding and spreading developed faster and became more prominent when the endogenous α-syn level was increased, as demonstrated in slices from mThy-1-human-α-syn transgenic (ASO) pups, where cell body inclusions were detected at the CA1 region by 7 dpi (Fig. 2b-d). The overexpression of α-syn in itself did not result in any aggregation in slices from ASO pups, as controlled for using PBS-injected OHSCs (Additional file 5: Figure S5).

Microinjection of PFFs was essential for the ordered inter-neuronal spread of PFF-templated aggregation from DG to CA3 and CA1, as application of PFF solution to the surface of the slice solely resulted in development of pS129-positive structures in the periphery of the slice (Additional file 6: Figure S6). This pattern is most likely caused by fluid flow across the slice surface.

**Fig. 3:**
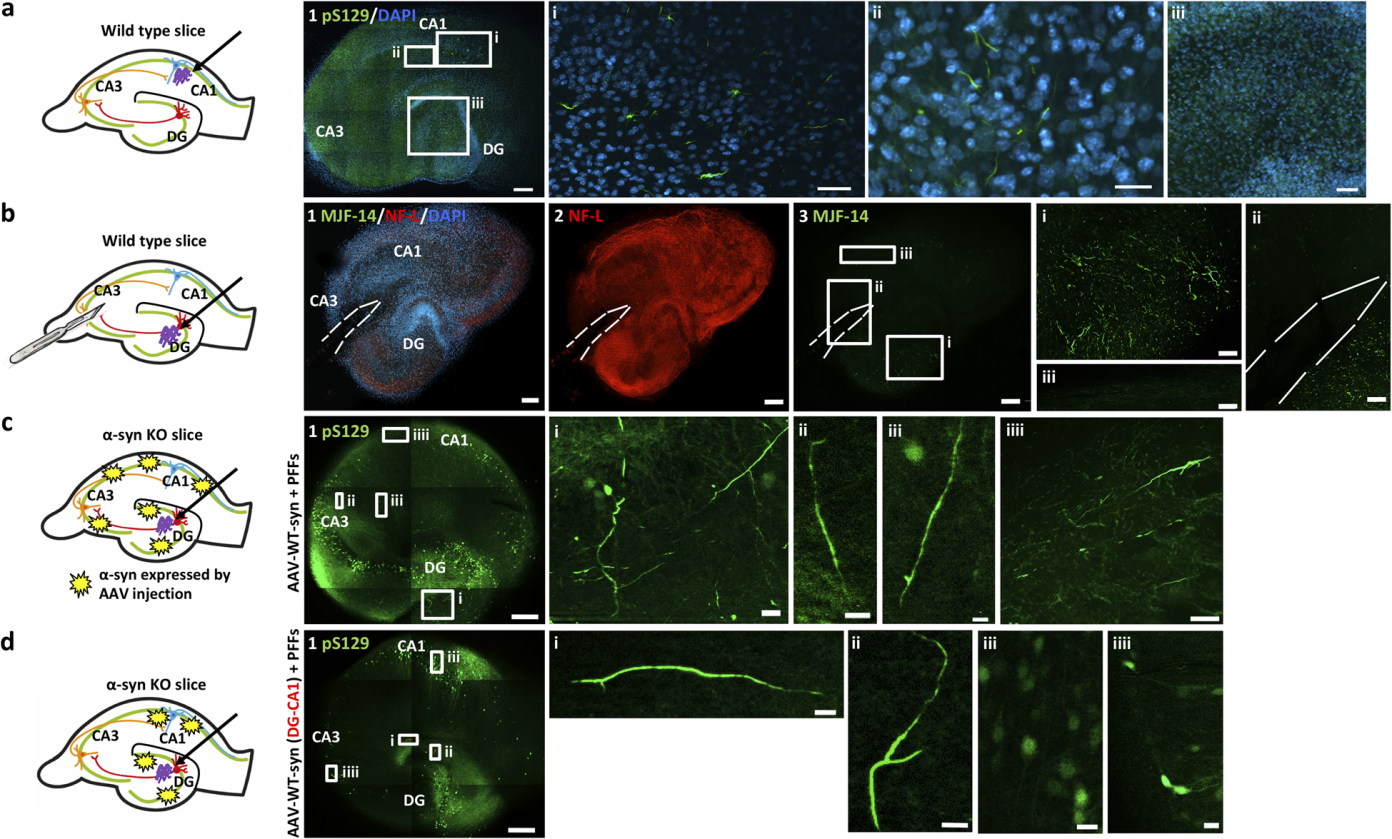
Application I. Demonstrating trans-synaptic spreading as a route for spreading of α-syn-aggregate pathology from DG via CA3 to the CA1 region using surgical and viral transgene methods. **a** Illustration of PFF injection in CA1 in WT OHSCs to test the efficiency of the retrograde route of spreading. **1** Composite image 14 dpi of S129A PFFs at CA1. Scale bar: 200 μm. MJF-14-positive aggregates are seen at the CA1 region (**i, ii**), but there is no spreading to DG (**iii)**. Scale bar i & iii: 50 μm, ii: 20 μm. **b** Diagram showing transection of axonal projections between DG and CA3, which blocks spreading of α-syn aggregate pathology from DG to CA1. The surgical destruction of the tissue is demonstrated by the absence of nuclei (**1**), axonal marker NF-L (**2**), and MJF-14 staining (**3**). Scale bars: 200 μm. Magnified images from **3** show aggregates at DG (**i**) and proximal to the cut (**ii**), but not distal to the lesion (**ii, iii**). Scale bars: 50 μm. **c** Diagram showing expression of WT**-**α-syn in α-syn KO slices by AAV vectors injected in DG, CA3, and CA1. **1** α-syn expression in DG, CA3, and CA1 supports spreading of aggregated pS129 α-syn (11A5) to CA1 7 dpi of PFFs in DG, as seen from the magnified panels **i**-**iiii**. Scale bar: 200 μm, i & iiii: 20 μm, ii & iii: 10 μm. Note the strong AAV-dependent expression of pS129 in some neuronal nuclei. **d** Illustration of WT**-**α-syn expression in DG and CA1 only of α-syn KO slices. **1** Absence of α-syn expression in the CA3 abolishes spreading of aggregation to CA1 at 7 dpi. Scale bar: 200 μm. **i, ii** pS129-positive aggregates are detectable at DG. **iii N**o pS129-positive aggregates are found at the CA1 region. **Iiii** A few neurons show nuclear expression of pS129-α-syn at CA3. Scale bars: i: 20 μm, ii, iii & iiii: 10 μm. Data in **a** are illustrative of 12 slices divided over 3 experiments. Images in **b** are representative of 4 experiments with 18 slices in total, while **c** & **d** representative of 3 separate experiments/18–21 slices in total per condition

Having established that seeded α-syn aggregate pathology can spread anterogradely from the DG to the CA1 region in the hippocampal slice model, we wanted to determine if retrograde spreading from the CA1 to the DG region could also occur. When slices were injected with S129A PFFs in the CA1 region, pS129-positive aggregates were observed at CA1 after 14 days, but no aggregates were found at the DG (Fig. 3a). Thus, at a time point twice as long as the one necessary for anterograde spreading to occur, no retrograde spreading is observed in the slice model.

To test whether the identified inter-neuronal spreading is trans-synaptic in the slice model, a surgical cut was made in the region between DG and CA3 through the whole thickness of the slice immediately after PFF injection. This would block the axonal transport required for spreading through the synaptic connections linking DG and CA3 regions. Loss of axonal connectivity between DG and CA3 in the slice blocked the formation of the MJF-14- and pS129-positive aggregates distal to the lesion compared with control slices with an intact connection through the DG-CA3-CA1 regions (Fig. 3b). This confirmed that synaptic connectivity between neurons was a necessity for spreading of aggregation but did not answer the question of true trans-synaptic spreading. In order to truly characterize the spreading as trans-synaptic would mean that the spreading of templated α-syn aggregate pathology requires expression of endogenous α-syn in all the connected neurons, as it is needed to amplify the small amount of seeds taken up by receiving neurons to facilitate further release of new seeds to the next neuron in the pathway.

To investigate this hypothesis, we used α-syn KO slices that by themselves do not allow development of pathology upon PFF injection (Fig. 2a,ii). In these slices, human α-syn was then expressed in neurons under the control of a synapsin-1 promoter using an AAV vector in either all regions of the OHSC or only at DG and CA1 regions (Fig. 3c, d). Successful expression was confirmed by immunostaining of AAV-injected slices using total α-syn (MJFR1), aggregated α-syn (MJF-14) and pS129 antibodies. Targeted regions displayed a decent α-syn staining as demonstrated by MJFR1-staining 10 days post transfection (Additional file 7: Figure S7a, b). When transfection was limited to the DG and CA1, α-syn expression was nicely restricted to these areas, with only a punctate staining visible in the proximal CA3 region, most likely corresponding to synaptic terminals of DG granule cells (Additional file 7: Figure S7c). At a closer look, the virally expressed α-syn appears to undergo systematic sorting in the neuron, albeit with a higher degree of cell body staining than in WT OHSCs, as anticipated from the viral overexpression (Additional file 7: Figure S7d). No aggregation was induced by the AAV-expression of α-syn in itself, as seen by the lack of staining using MJF-14 and pS129 (Additional file 8: Figure S8a, b). Some pS129-positivity was detected as a diffuse staining in the cell bodies and nuclei of AAV-WT-α-syn transfected neurons in primarily the CA regions, especially at 17 days post transfection (21 DIV) (Additional file 8: Figure S8c).

Injection of α-syn-expressing vectors in the DG, CA3, and CA1 regions of the α-syn KO slice combined with PFF injection in the DG allowed inter-neuronal spreading of α-syn aggregate pathology to the synaptically connected CA3 and CA1 regions (Fig. 3c) comparable to the data generated in wild type hippocampal slices (Fig. 2b). The pathology progressed further at 14 dpi, with perinuclear pS129-positive aggregates found in the CA1 region (Additional file 9: Figure S9a), as in the case of wild type slices 14 dpi of PFFs (Additional file 3: Figure S3).

In contrast, when the synaptic circuit was left without α-syn expression at CA3, in order to disrupt the hypothesized sequential trans-synaptic spreading, no spreading to the CA1 region was detectable at 7 dpi of S129A PFFs in the DG (Fig. 3d). Only a faint nuclear pS129-staining was seen in the CA1 region (Fig. 3,iii & iiii), despite substantial aggregation in the DG (Fig. 3d,i & ii). Even at 14 dpi, no pathology was detectable in the CA1 region, which only displays diffuse nuclear staining (Additional file 9: Figure S9b,iii). These results strongly support the hypothesis of sequential trans-synaptic spreading of α-syn aggregate pathology.

### Application 2: using OHSCs from α-syn KO mice and AAV vectors to demonstrate that α-syn aggregation and spreading occurs independently of its phosphorylation at serine-129

**Fig. 4:**
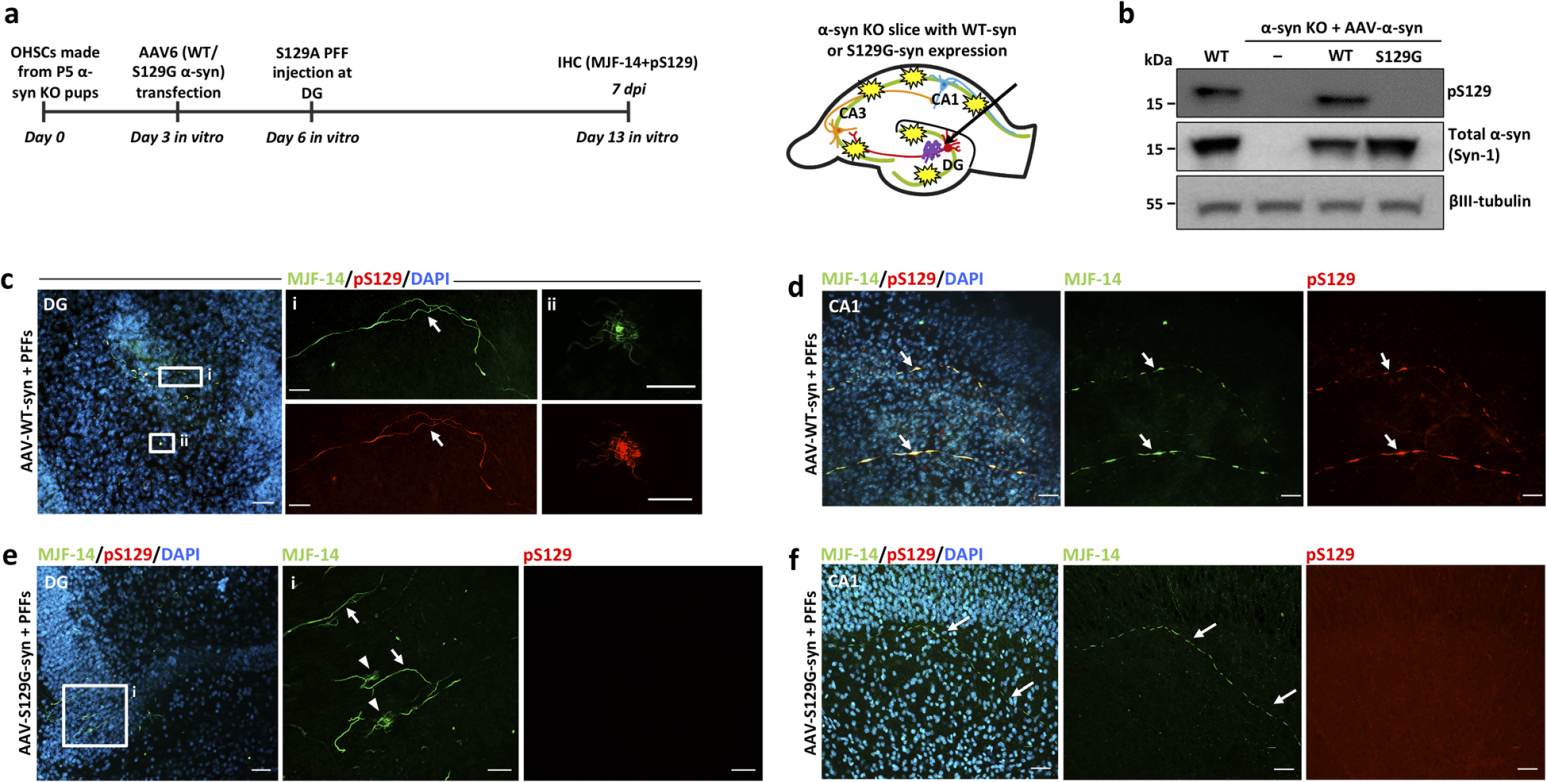
Application II. Demonstrating that phosphorylation of S129 on α-syn is not a prerequisite for seeding α-syn aggregation or trans-synaptic spreading in hippocampal slices. **a** Experimental setup with establishment of neuronal expression of either WT- or non-phosphorylatable S129G-α-syn in α-syn KO slices prior to initiation of templated α-syn aggregation by injection of S129A PFFs. **b** Validation of virally mediated WT- and non-phosphorylatable S129G-α-syn expression in α-syn KO slices using antibodies against total and pS129-α-syn (11A5). **c** Expression of WT α-syn supports establishment of MJF-14- and pS129-positive (11A5) aggregate pathology in the DG region following PFF injection at DG. Magnified panels show axonal aggregates (**i**) and cell body inclusions (**ii**) at DG. Scale bar: 50 μm, **i**: 20 μm, **ii**: 5 μm. **d** MJF-14- and pS129 positive (11A5) pathology spreads to the CA1 region within 7 dpi. Scale bar: 50 μm. **e** Expression of S129G-α-syn supports establishment of MJF-14-positive/pS129-negative aggregate pathology in the DG, present in axons (**i**, arrows) and cell bodies (**i**, arrowheads). Scale bar: 50 μm, **i**: 20 μm. **f** The non-phosphorylated MJF-14-positive aggregate pathology spreads to the CA1 region within 7 dpi. Scale bar: 50 μm. Western blot in **b** is representative of 3 independent experiments, while images in **c**-**f** are illustrative of 3–5 experiments with 21–30 slices in total per condition

Phosphorylation at serine-129 in α-syn is characterized as a hallmark of pathological α-syn inclusions in the human brain. However, its pathophysiological role in relation to aggregation and cytotoxicity has been contested [19, 37, 38]. To investigate the role of this post-translational modification in the trans-synaptic spreading of seeded α-syn aggregation, we expressed either non-phosphorylatable S129G-mutated α-syn or WT α-syn in α-syn KO slices using AAV vectors. After injection with S129A PFFs, we compared the spreading of MJF-14-positive aggregates for both variants (Fig. 4a). S129G- and WT-α-syn were expressed at comparable levels in the α-syn KO slices as determined by immunoblotting, but only the WT protein was phosphorylated at serine-129 (Fig. 4b). The absence of a positive band in the homogenate from control non-injected α-syn KO slices confirmed the specificity of the pS129 antibody used in the experiment (Fig. 4b). Both transgenic variants of α-syn supported the development of MJF-14-positive aggregates in the DG regions (Fig. 4c, e) and spreading to the CA1 region (Fig. 4d, f), when S129A PFFs were injected into the DG. However, only the WT form of α-syn was phosphorylated at S129 (compare Fig. 4c, d & e, f). The S129-phosphorylated and non-phosphorylatable aggregates appeared comparable with respect to localization in both axons and cell bodies. Consequently, we concluded that phosphorylation at S129 in α-syn is not critical for aggregate formation or inter-neuronal spreading thereof.

The organotypic hippocampal slice model is thus a versatile tool that allows investigation of the role of site-specific post-translational modifications in spreading of seeded α-syn in a time and cost scale that is much smaller than developing a transgenic animal model on an α-syn KO background.

## Discussion

The correlation of PD symptomatology with progressive accumulation of the pathological α-syn aggregate-containing Lewy bodies in various brain regions formed the basis for the Braak hypothesis [1]. The hypothesis was corroborated by the demonstration of Lewy body pathology in fetal neurons transplanted into the striatum of PD patients 11–16 years prior to their death [39]. Since then, the general hypothesis of a prion-like spreading of templated misfolded proteins – already comprising diseases like Creutzfeldt-Jakob disease and Alzheimer’s disease – was expanded to include other diseases such as PD, dementia with Lewy bodies (DLB), and multiple system atrophy (MSA), where seeds of aggregated α-syn are considered the culprit [11, 40, 41]. Still, creating models suitable for the investigation of the hypothesized prion-like spreading of α-syn has not been easy. In vivo models of seeded α-syn aggregation are slow and costly, while in vitro cell-based models developed to facilitate mechanistic investigations and drug screening lack the complexity of the living brain [42–44].

In this study, we present a novel ex vivo brain tissue culture model that can serve as a translational step between cell-based in vitro and in vivo models for synucleinopathies. The model takes advantage of the organotypic hippocampal slice culture that comprises a well suited tissue setup, which has been used for electrophysiological studies for decades [34]. For one thing, it possesses three synaptically connected neuronal populations located in a developmentally evolved brain structure. Moreover, the neurons are embedded in an active matrix of glia cells, and the model thus outperforms currently available two- and even three-dimensional in vitro culture systems [43, 45]. The aggregation in the model is based on microinjection of α-syn PFFs into the DG, which seed a progressive, templated aggregation of endogenous mouse α-syn that spreads through CA3 to CA1 neurons, and thus allows efficient study of endogenous aggregated structures. Application of the PFFs by microinjection is critical for the model as it induces aggregation and spreading in the interconnected neurons of the DG, CA3, and CA1 circuit, whereas simple application of PFFs on top of the slice only induces development of aggregate-containing neurons in the periphery of the slice. The explanation for these distinct aggregation patterns might stem in part from fluid flow towards the edges of the domed OHSC after drop application and in part from the fact that glial scar formation on top of the slice reduces accessibility of neurons upon drop application compared to injection [46]. Thus, a greater proportion of the applied PFFs are likely taken up by astrocytes and microglia and degraded, as seen in previous studies [47, 48].

The use of S129A PFFs, which cannot be phosphorylated at S129, and detection of cellular aggregates by pS129-α-syn antibodies allows the unequivocal detection of de novo formed inclusions in the tissue and not merely the injected material. Comparison of aggregation induced by the injection WT PFFs versus S129A PFFs in the OHSCs resulted in similar timing and pattern of the aggregates, thus justifying the use of the S129A PFFs. The inclusions in the model share the disease-associated epitopes with aggregates present in human brains affected by synucleinopathies, as demonstrated by the binding of both pS129-α-syn antibodies and the conformation-specific α-syn aggregate antibody MJF-14 [49]. The PFF-induced inclusions demonstrate various patterns that are topology- and time-dependent. The aggregates observed at DG by 7 and 14 dpi resemble filamentous structures that wrap the nucleus, while more compacted, often spherical, cytoplasmic inclusions develop in the CA1 region at 14 dpi, suggesting the development of Lewy body-like inclusions in this region. This is in agreement with previous findings establishing that Math2-expressing neurons in the CA1 region are more affected by α-syn pathology following seeding with PFFs in vivo compared to DG neurons, which are relatively spared [50].

When PFFs are injected in α-syn KO slices, no pathology develops, demonstrating that neuronal α-syn expression is essential for the templating of α-syn aggregate pathology in the model, in accordance with previous in vivo models [13]. The experiments done to track the injected PFFs in the α-syn KO slices showed that the PFFs undergo truncation at the C-terminal but stay in the tissue for at least 7 days following the injection, in line with previously reported data [16, 51]. Efforts to determine cellular localization of the injected PFFs over time in the model using the available antibodies have yet to prove successful and need further optimization.

Looking at overexpression conditions, aggregation and spreading of α-syn after PFF injection is enhanced in slices made from α-syn over expressing pups (mThy-1-human α-syn-expressing mice) compared to wild type OHSCs. This is in agreement with the previous finding that development and progression of α-syn pathology in humans depends on the level of α-syn expression, with faster disease progression in PD patients with duplications or triplications of the SNCA gene [52, 53]. No aggregation resulted from the overexpression of α-syn in itself.

Whether there is a preferential directionality of α-syn pathology spreading remains unclear due the varying results observed after inoculation of seeds in the striatum, hippocampus or olfactory bulb [54, 55]. A component of retrograde axonal transport is suggested to transfer seeds from the gut to the vagal nucleus, as demonstrated in rodents [56], but anterograde transport has also been observed using reconstructed human neuronal networks [42]. Furthermore, bidirectional propagation was recently reported after detection α-syn pathology in both cardiac and gastric tissue following PFF seeding in the duodenum of a rat model [57]. Here, we used the organotypic slice model to address the question of a preferred direction of spreading of templated aggregation in hippocampal tissue by comparing the spreading upon injection of PFF into either the DG or the CA1. Anterograde transfer from the DG to the CA1 region appeared to be the preferred mode, as PFF injection in DG induced α-syn aggregation and spreading to the CA1 region within 7 days. In contrast, injection of PFFs in CA1 only triggered α-syn aggregation at the site of injection, even incubating slices 14 days after inoculation.

The lack of spreading in the retrograde direction is somewhat surprising, considering the amount of studies, in which retrograde spreading has previously been demonstrated [56, 58–60]. However, it has recently been shown that neural connectivity by itself cannot fully explain the pattern of pathology in PD and models thereof, and that other factors, including α-syn expression levels, play a crucial role [58]. Furthermore, a variety of mechanisms have been shown to potentially contribute to both the release and uptake of aggregated α-syn species, including exosomes, misfolding-associated protein secretion, tunneling nanotubes, endocytosis, binding to specific receptors such as LAG-3 etc. [42, 61, 62]. Thus, discrete distinctions in the prevalence and utilization of some of these mechanisms, or in the expression of contributing proteins, between brain regions could explain the dissimilarities in spreading directions in various models of synucleinopathies. Even within the hippocampal formation, several discrete neuronal populations with specific protein expression profiles exist [63], likely characterized by differences in mechanisms used to cope with protein aggregation. By way of example, we observed distinct morphologies of α-syn cell body aggregates in the hippocampal subregions in our model, possibly reflecting differential handling of the aggregates by the various neuronal subtypes. In this case, the striking spherical compaction of cell body aggregates in the CA1 region compared to the DG might translate into a decreased release of α-syn seeds into the extracellular space around these neurons, thereby minimizing retrograde spreading after injection of PFFs in CA1. Additionally, whether differential gene expression in the neuronal populations might result in a poor ability of CA3 neurons to take up aggregated α-syn species at their terminals is unknown. Furthermore, a recent review points toward the CA2 subregion of the hippocampus, located in-between CA3 and CA1, as an underrated area in PD and related disorders [64]. The CA2 pyramidal neurons display distinctive characteristics regarding both protein expression and physiology, including synaptic function, and might thus play an important role in the spreading of pathology – or lack thereof – between hippocampal subregions [64].

The hypothesis of trans-synaptic spreading in the slice model was corroborated by the fact that cutting the axons in the CA3 region blocked spreading to regions distal to the lesion. To demonstrate that the spreading of pathology not only requires an intact synaptic connection but also contiguous α-syn expression, we generated hippocampal slices from α-syn KO pups in which neuronal α-syn expression was established locally using an AAV vector expressing α-syn. These slices were able to support templated spreading from DG to CA1 when AAV-mediated α-syn expression was established in all connected regions, i.e. DG, CA3, and CA1. Conversely, no spreading occurred when the slices lacked α-syn expression at the CA3 region. In some instances, we did see a few cell bodies in the CA3 region displaying pS129-staining, even though AAV vectors had not been injected in this region. This could either reflect a small degree of retrograde transfer of the rAAV2/6 after uptake at the terminals of the CA3 neurons, as has previously been demonstrated [65], or an inter neuronal transfer of α-syn from DG or CA1 neurons, in line with another finding [66]. However, in no case was this minor α-syn expression at the CA3 enough to support spreading of aggregation. In conclusion, this experiment demonstrates that aggregation in the CA1 region is due to the transfer of templated pathological α-syn aggregates through the synaptic circuit, requiring renewed templating in each recipient population and not just transfer of injected PFFs through the neuronal circuit.

The viral vector approach to expression and silencing of genes of interest makes the organotypic slice model a versatile tool for constructing tailor-made slices where candidate genes are modulated. As proof of concept, we asked if phosphorylation at S129 is necessary for seeded α-syn aggregation and spreading. Phosphorylation at S129 represents the most abundant post-translational modification of α-syn in Lewy bodies [29]. This phosphorylation has received much attention in the last decade [67], as pS129-targeting antibodies have so far been considered the best tool to demonstrate abnormal α-syn deposits in brain tissue. Moreover, the modification has been suggested to contribute to α-syn cytotoxicity [19, 21], although this claim is contested by others [37, 38, 68, 69]. To address the question of the role of pS129 in aggregation and spreading, we used AAV vectors to generate expression of non-phosphorylatable S129G-mutant human α-syn or WT human α-syn in α-syn KO slices. The production of slices solely expressing S129G α-syn allowed us to conclude that phosphorylation at S129 is not a prerequisite for initiation of α-syn aggregation or its inter-neuronal spreading following the PFF injection. The approach of transgenic expression of α-syn species in KO tissue can be extended to investigate the role of other post-translational α-syn modifications like truncations, ubiquitinations on specific lysines, or N-terminal acetylations, as well as for validating proteins involved in the spreading process. The slice model is an innovative alternative to transgenic animals, being significantly easier, faster, and less costly; most importantly, it is in accordance with the “3R” concept values of replacement, reduction and refinement, regarding the use of animals in experiments.

Naturally, the model is not without limitations; firstly, inter-individual variations between mice and anatomic variations among sectioned slices throughout the hippocampus can pose a challenge. However, the hippocampi of one pup generally yield around 8–18 suitable slices, allowing both the generation of almost identical tissue pools for paired experimental conditions and the use of multiple slices per experiment. Combined with a careful selection and division of slices between groups, the influence of slice and mouse variations in the final results can be minimized. Secondly, the fact that only sparse aggregation is induced by PFF injection in wild type slices complicates biochemical studies of e.g. toxicity of aggregates. Conversely, the sparseness may be regarded as an advantage for studying the effect of different α-syn aggregate strains on uptake, seeding, and spreading. It might also facilitate research into factors governing selective neuronal vulnerability and thus the model resembles in vivo conditions in the sick brain, where only select, vulnerable neurons rather than the whole population display synuclein pathology.

## Conclusion

This study presents a novel ex vivo brain tissue model for studying seeded α-syn aggregation and inter-neuronal spreading in circuitry-connected neurons through the use of organotypic hippocampal slices. The model is superior to previous in vitro models with regard to replicating the hypothesized pathophysiological neuronal handling of α-syn aggregates, as only the first, recipient neurons in DG are exposed to in vitro formed aggregates. The subsequent spreading of seeding-competent species represents novel *in cellulo*-generated aggregates, as demonstrated by the absence of aggregates in slices from α-syn KO mice and the absence of spreading from DG to CA1 when no α-syn is expressed in the CA3 neurons.

With respect to post-translational modifications, we established that phosphorylation at S129 is not a necessity for aggregation or spreading in the slice model. The model provides opportunities for novel, attractive, and beneficial applications in the field of synucleinopathies, based on the use of various genetic mouse lines and methods such as viral vector-based gene regulation, super-resolution microscopy, live imaging, electrophysiological recordings, and pharmacological treatment.

## Supplementary information

**Additional file 1: Figure S1.** Characterization of the pre-formed fibrils used to initiate intra-neuronal α-syn aggregation upon injection into the OHSC. **a, b** Biochemical characterization of S129A (**a**) and WT (**b**) PFFs. The insoluble fibrils consist of pure α-syn with negligible fragmentation as demonstrated by SDS-PAGE and Coomassie blue staining (**i**). Molecular size markers in kDa are indicated. **ii**: The amyloid nature of the PFFs was confirmed by a robust K114 fluorometric signal detected at 550 nm compared to the absence of signal for monomeric S129A or WT α-syn. **iii**: The sonicated S129A and WT PFFs comprise homogeneous, mono-dispersed particle populations with a 44 nm (**a**) or 38 nm (**b**) hydrodynamic radius as determined by DLS. **c** OHSCs from α-syn KO pups were injected with S129A PFFs and tissue extracted at 2 h post injection (hpi), 3 and 7 dpi in 4% SDS/7 M urea to study the fate of injected PFFs. The depolymerized PFFs were probed with antibodies targeting either the C-terminal (MJFR1) or amino acid residues 91–99 (Syn-1), demonstrating the progressive disappearance of intact α-syn (approx. 16 kDa) with complete loss after 7 dpi. The Syn-1 antibody, however, also detects a C-terminally truncated species (approx. 12 kDa) that remains in the tissue for more than 7 dpi. Molecular size markers (kDa) are indicated. **d** Composite images of α-syn KO slices injected with S129A PFFs. Immunostaining using the MJFR1 antibody showed a dome-shaped signal at the site of injection at DG at 2 hpi, while the signal had disappeared at 7 dpi, supporting the C-terminal truncation of injected material within this timeframe. Scale bar: 200 μm. Western blot data in **c** are illustrative of 3 independent experiments, while images in **d** are representative of 2 separate experiments/6 slices in total per time point.

**Additional file 2: Figure S2.** Injection of WT α-syn PFFs in WT OHSCs results in the formation of endogenous α-syn aggregates with the same timing and morphology as injection with S129A-mutated PFFs. **a** At 3 dpi, small serpentine aggregates start to appear at the DG (**i**, arrows), which increase in size at 5 dpi (**ii**, arrows). At 7 dpi, cell body aggregates emerge in the DG (**iii**, arrowheads). Scale bars i & ii: 20 μm, iii: 50 μm. **b** Aggregation spreads to the CA1 around 7 dpi where axonal aggregates become visible (arrows). Scale bar: 50 μm. Images are representative from 2 to 4 experiments with a total of 5–15 slices per time point.

**Additional file 3: Figure S3.** OHSC injected with S129A PFFs at the DG and incubated for 14 days before immunostaining for aggregated α-syn (MJF-14, green), pS129-α-syn (11A5, red) and nuclei (DAPI, blue). Scale bar: 200 μm. Panels **i**, **ii** and **iii** represent merged high-magnification images of aggregated (MJF-14, green) and pS129-α-syn (11A5, red) from DG (**i**), CA3 (**ii**), and CA1 regions (**iii**). Arrows designate axonal aggregates and arrowheads illustrate nuclear inclusions. Scale bars: 20 μm. Images are representative of 3 separate experiments with 13 slices in total.

**Additional file 4: Figure S4:** Region- and time-dependent development of various α-syn inclusion patterns. **a** 7 to 10 dpi of PFFs, the pS129-positive α-syn aggregates (11A5) present as filamentous structures that surround the DAPI-stained nuclei. Scale bars: 10 μm. **b** 14 dpi of PFFs, the pS129-positive aggregates at DG are still filamentous, while inclusions at CA regions, mainly at CA1, present as spherical, denser cytoplasmic inclusions resembling Lewy bodies. Scale bars: 10 μm. Representative images from minimum 13 slices/3 separate experiments per time point.

**Additional file 5: Figure S5.** Transgenic overexpression of α-syn does not induce aggregation. mThy-1-human-α-syn transgenic OHSCs injected with PBS do not display any aggregation as detected by MJF-14 (green) and pS129 (11A5, red) staining. Only a weak pS129-staining is seen in the cell bodies of the hippocampal neurons. Scale bar: 200 μm, insets: 50 μm. Illustrative images from 3 slices.

**Additional file 6: Figure S6.** Composite image of WT OHSC with S129A PFFs applied as a drop on the surface of the slice 7 days post application, showing pS129-positive aggregates (D1R1R) found only at the periphery of the slice. Scale bar: 200 μm. Magnified images show the aggregates at the periphery of the slice in CA1 (**i**) and CA3 (**ii**). No aggregates were detected at DG region (**iii**). Scale bars for i & ii: 50 μm, iii: 200 μm. Images are representative of 3 experiments/14 slices in total.

**Additional file 7: Figure S7.** AAV-construct injection results in ample α-syn expression. **a, b** At 10 days post injection of AAV-α-syn (corresponding to 14 DIV), both the WT variant (**a**) and the S129G variant (**b**) give rise to a robust α-syn expression in all transfected regions, as detected by total human α-syn antibody MJFR1 (green). Panels show magnified images from the DG (**i**), CA3 (**ii**) and CA1 (**iii**), displaying α-syn positive neurons. Scale bars: 200 μm, insets: 50 μm. **c** Transfection with AAV in only the DG and CA1 results in α-syn expression limited to these areas (**i**). In the CA3 region, the proximal part displays a punctate α-syn staining resembling synaptic terminals (**ii**, arrows), while the distal part is clear of staining (**ii**). No cell body staining in the CA3 is seen (**ii**). Arrowheads designate α-syn positive cell bodies. Scale bar: 200 μm, insets: 50 μm. **d** High magnification image showing the α-syn distribution inside a transfected neuron in the DG. The punctate staining indicates an efficient sorting of the expressed α-syn. Scale bar: 20 μm. Images are representative from 3 to 5 individual experiments with 13–20 slices per group.

**Additional file 8: Figure S8.** AAV-mediated overexpression of α-syn does not result in α-syn aggregation. **a, b** Staining for aggregated α-syn (MJF-14, green) and pS129-α-syn (11A5, red) at 10 days post transfection (14 DIV) does not detect any aggregation in either AAV-WT-α-syn (**a**) or AAV-S129G-α-syn slices (**b**). Insets show magnified images from DG (**i**), CA3 (**ii**) and CA1 (**iii**). Scale bars: 200 μm, insets: 50 μm. **c** At 17 days post transfection (21 DIV), a weak pS129-staining of particularly the pyramidal neurons of CA regions was seen in slices transfected with AAV-WT-α-syn. Magnified images show the co-localization of diffuse cell body pS129-staining with DAPI-stained nuclei. Scale bars: 200 μm, inset: 10 μm. Images are illustrative examples from 3 to 5 separate experiments/13–20 slices in total per group.

**Additional file 9: Figure S9. a** 14 dpi of S129A PFFs in AAV-WT-α-syn expressing slices, robust aggregation and spreading throughout the hippocampal slice is detected by pS129-staining (D1R1R). Panels show magnified images of aggregates from DG (**i**), CA3 (**ii**) and CA1 (**iii**). Scale bars: 200 μm, i: 20 μm, ii & iii: 10 μm. **b** At the same time in slices only expressing AAV-WT-α-syn in the DG and CA1, leaving the CA3 blank of expression, aggregation in the DG is seen (**i**), equal to the slices expressing α-syn throughout the circuit. However, no aggregation is seen in either CA3 (**ii**) or CA1 regions (**iii**). Scale bars: 200 μm, i: 20 μm, ii & iii: 10 μm. Representative images from 3 independent experiments with 12–16 slices per group.

## Data Availability

All data generated or analyzed during this study are included in this article.

## Abbreviations

AAV: Adeno-associated virus
α-syn KO: Alpha-synuclein knockout
α-syn: Alpha-synuclein
ASO: Alpha-synuclein over-expressing (mThy-1-human-α-syn transgenic)
BCA: Bicinchoninic acid assay
BSA: Bovine serum albumin
DAPI: 4′,6-diamidino-2-phenylindole
DG: Dentate gyrus
DIV: Days in vitro
DLB: Dementia with Lewy bodies
dpi: Days post injection
hpi: Hours post injection
ITR: Inverted terminal repeat
K114: (trans,trans)-1-bromo-2,5-bis-(4-hydroxy)styrylbenzene
MSA: Multiple system atrophy
OHSCs: Organotypic hippocampal slice cultures
PBS: Phosphate-buffered saline
PCR: Polymerase chain reaction
PD: Parkinson’s disease
PFA: Paraformaldehyde
PFFs: Pre-formed α-syn fibrils
pS129: Serine-129 phosphorylation
RIPA: Radioimmunoprecipitation assay
RT: Room temperature
SDS-PAGE: Sodium dodecyl sulphate polyacrylamide gel electrophoresis
TBS: Tris-buffered saline
WT: Wild type
