# Supplementary figures and images for: Organotypic slice culture model demonstrates inter-neuronal spreading of alpha-synuclein aggregates
Source: Acta Neuropathol Commun. 2019 Dec 19;7:213. doi: 10.1186/s40478-019-0865-5 (PMC6924077; doi:10.1186/s40478-019-0865-5)

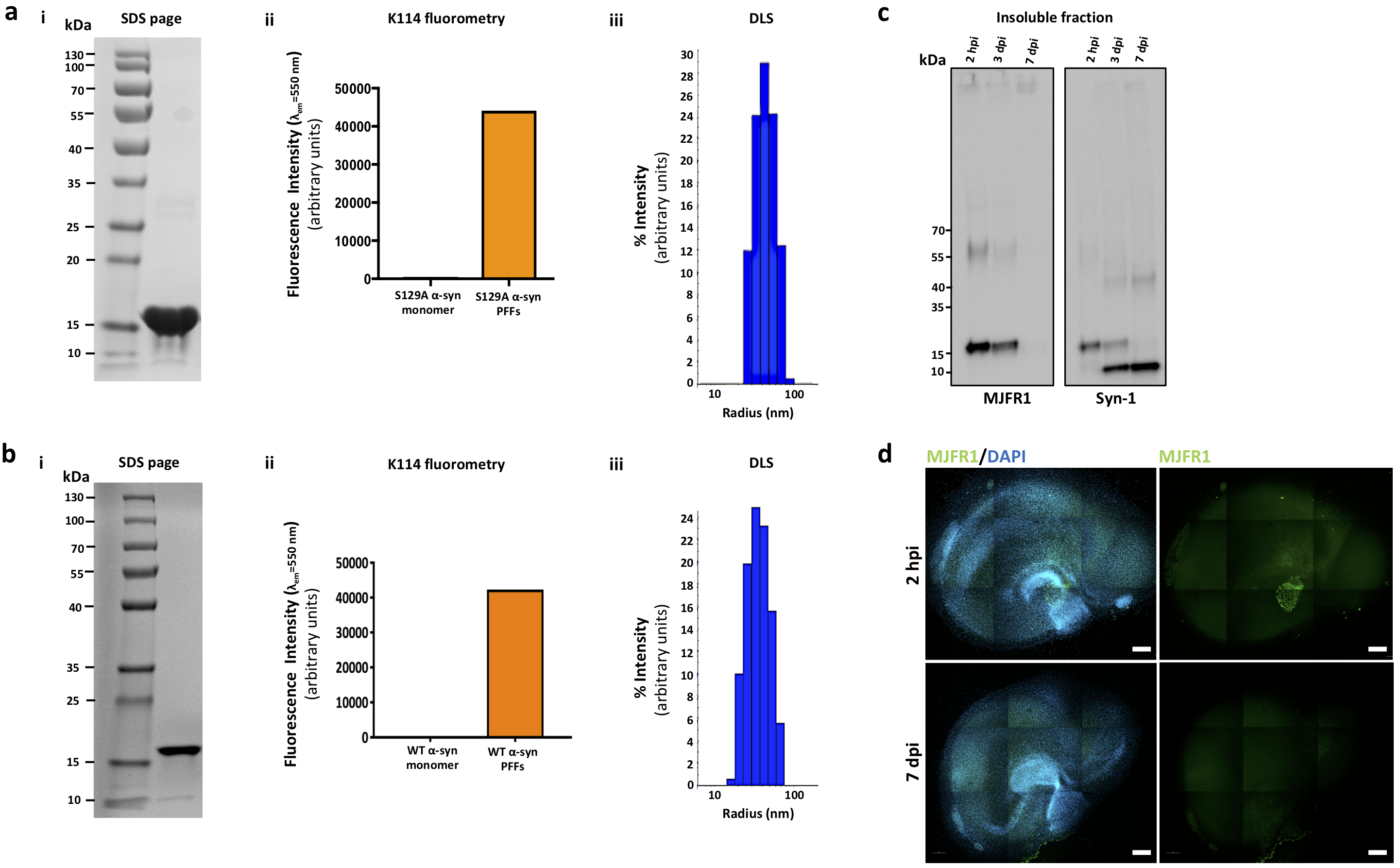

Supplement: Supplementary file 1 — Additional file 1: Figure S1. Characterization of the pre-formed fibrils used to initiate intra-neuronal α-syn aggregation upon injection into the OHSC. a, b Biochemical characterization of S129A (a) and WT (b) PFFs. The insoluble fibrils consist of pure α-syn with negligible fragmentation as demonstrated by SDS-PAGE and Coomassie blue staining (i). Molecular size markers in kDa are indicated. ii: The amyloid nature of the PFFs was confirmed by a robust K114 fluorometric signal detected at 550 nm compared to the absence of signal for monomeric S129A or WT α-syn. iii: The sonicated S129A and WT PFFs comprise homogeneous, mono-dispersed particle populations with a 44 nm (a) or 38 nm (b) hydrodynamic radius as determined by DLS. c OHSCs from α-syn KO pups were injected with S129A PFFs and tissue extracted at 2 h post injection (hpi), 3 and 7 dpi in 4% SDS/7 M urea to study the fate of injected PFFs. The depolymerized PFFs were probed with antibodies targeting either the C-terminal (MJFR1) or amino acid residues 91–99 (Syn-1), demonstrating the progressive disappearance of intact α-syn (approx. 16 kDa) with complete loss after 7 dpi. The Syn-1 antibody, however, also detects a C-terminally truncated species (approx. 12 kDa) that remains in the tissue for more than 7 dpi. Molecular size markers (kDa) are indicated. d Composite images of α-syn KO slices injected with S129A PFFs. Immunostaining using the MJFR1 antibody showed a dome-shaped signal at the site of injection at DG at 2 hpi, while the signal had disappeared at 7 dpi, supporting the C-terminal truncation of injected material within this timeframe. Scale bar: 200 μm. Western blot data in c are illustrative of 3 independent experiments, while images in d are representative of 2 separate experiments/6 slices in total per time point. [file 40478_2019_865_MOESM1_ESM.tiff]

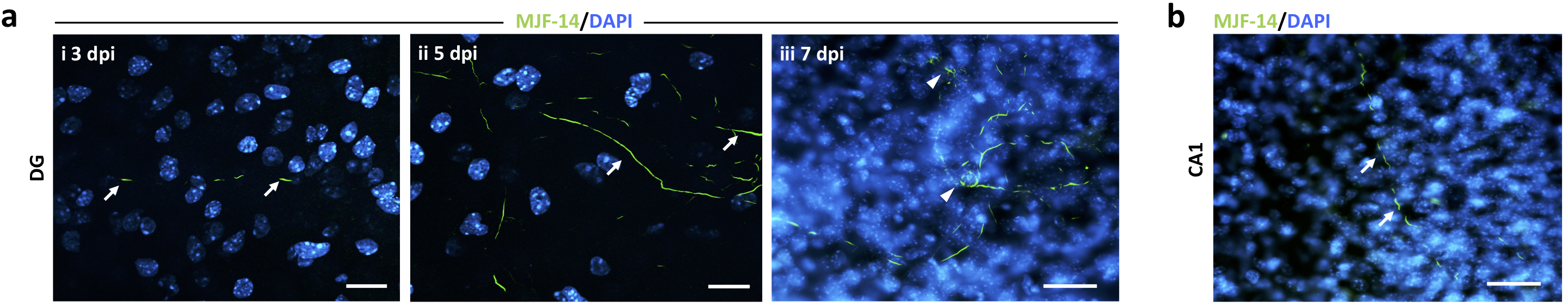

Supplement: Supplementary file 2 — Additional file 2: Figure S2. Injection of WT α-syn PFFs in WT OHSCs results in the formation of endogenous α-syn aggregates with the same timing and morphology as injection with S129A-mutated PFFs. a At 3 dpi, small serpentine aggregates start to appear at the DG (i, arrows), which increase in size at 5 dpi (ii, arrows). At 7 dpi, cell body aggregates emerge in the DG (iii, arrowheads). Scale bars i & ii: 20 μm, iii: 50 μm. b Aggregation spreads to the CA1 around 7 dpi where axonal aggregates become visible (arrows). Scale bar: 50 μm. Images are representative from 2 to 4 experiments with a total of 5–15 slices per time point. [file 40478_2019_865_MOESM2_ESM.tiff]

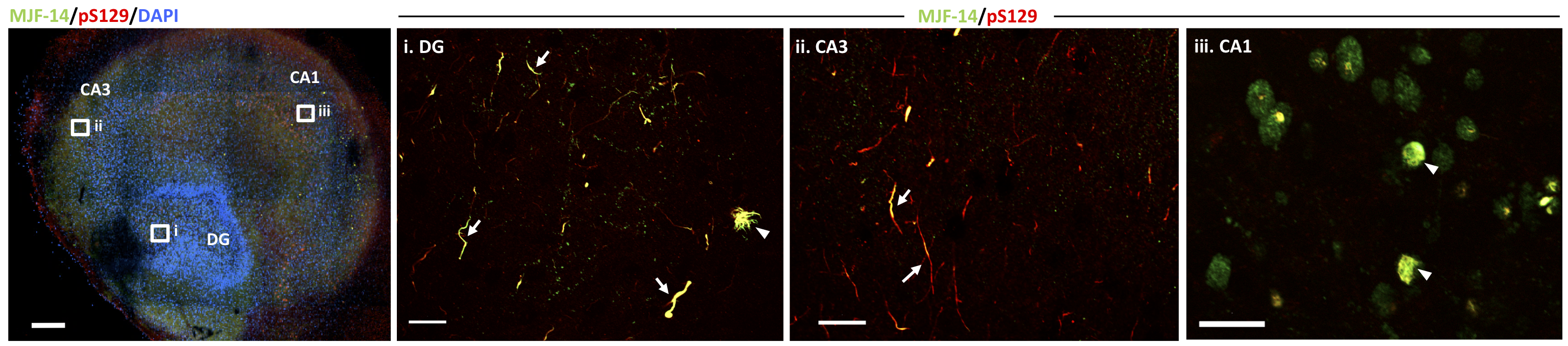

Supplement: Supplementary file 3 — Additional file 3: Figure S3. OHSC injected with S129A PFFs at the DG and incubated for 14 days before immunostaining for aggregated α-syn (MJF-14, green), pS129-α-syn (11A5, red) and nuclei (DAPI, blue). Scale bar: 200 μm. Panels i, ii and iii represent merged high-magnification images of aggregated (MJF-14, green) and pS129-α-syn (11A5, red) from DG (i), CA3 (ii), and CA1 regions (iii). Arrows designate axonal aggregates and arrowheads illustrate nuclear inclusions. Scale bars: 20 μm. Images are representative of 3 separate experiments with 13 slices in total. [file 40478_2019_865_MOESM3_ESM.tiff]

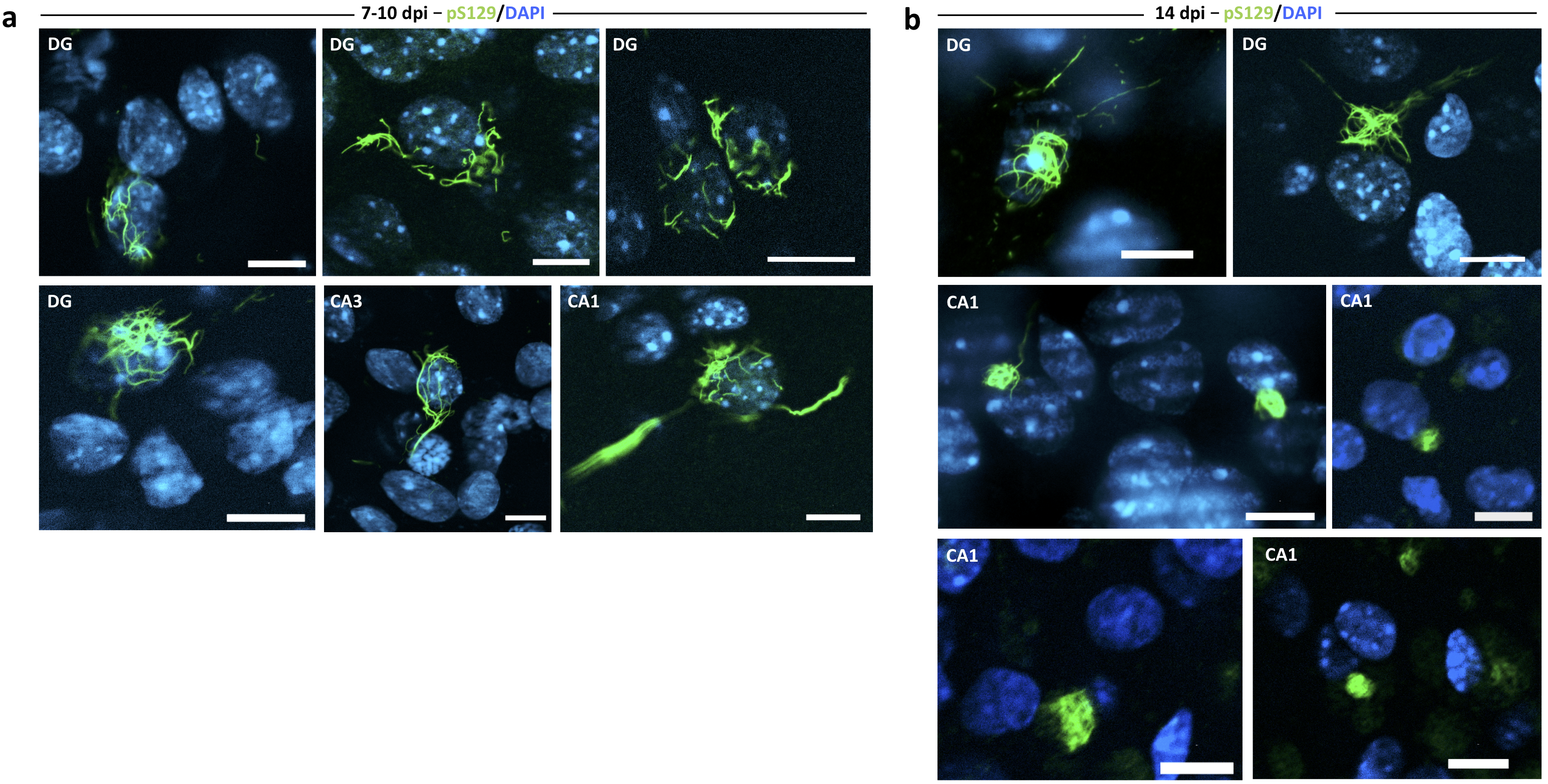

Supplement: Supplementary file 4 — Additional file 4: Figure S4: Region- and time-dependent development of various α-syn inclusion patterns. a 7 to 10 dpi of PFFs, the pS129-positive α-syn aggregates (11A5) present as filamentous structures that surround the DAPI-stained nuclei. Scale bars: 10 μm. b 14 dpi of PFFs, the pS129-positive aggregates at DG are still filamentous, while inclusions at CA regions, mainly at CA1, present as spherical, denser cytoplasmic inclusions resembling Lewy bodies. Scale bars: 10 μm. Representative images from minimum 13 slices/3 separate experiments per time point. [file 40478_2019_865_MOESM4_ESM.tiff]

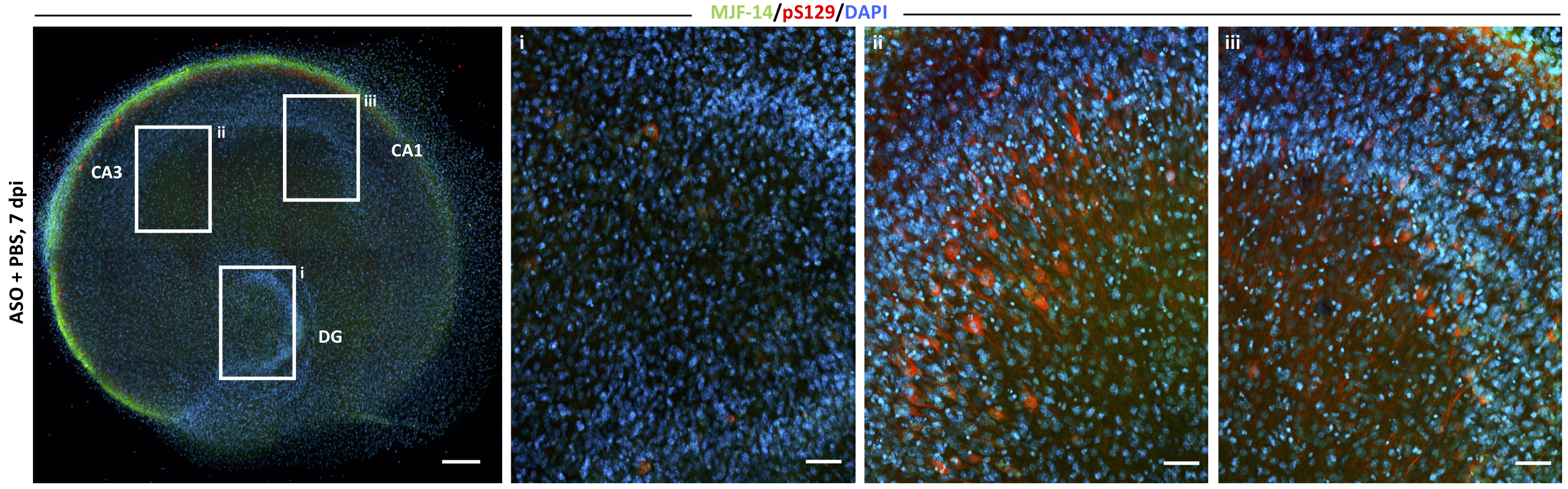

Supplement: Supplementary file 5 — Additional file 5: Figure S5. Transgenic overexpression of α-syn does not induce aggregation. mThy-1-human-α-syn transgenic OHSCs injected with PBS do not display any aggregation as detected by MJF-14 (green) and pS129 (11A5, red) staining. Only a weak pS129-staining is seen in the cell bodies of the hippocampal neurons. Scale bar: 200 μm, insets: 50 μm. Illustrative images from 3 slices. [file 40478_2019_865_MOESM5_ESM.tiff]

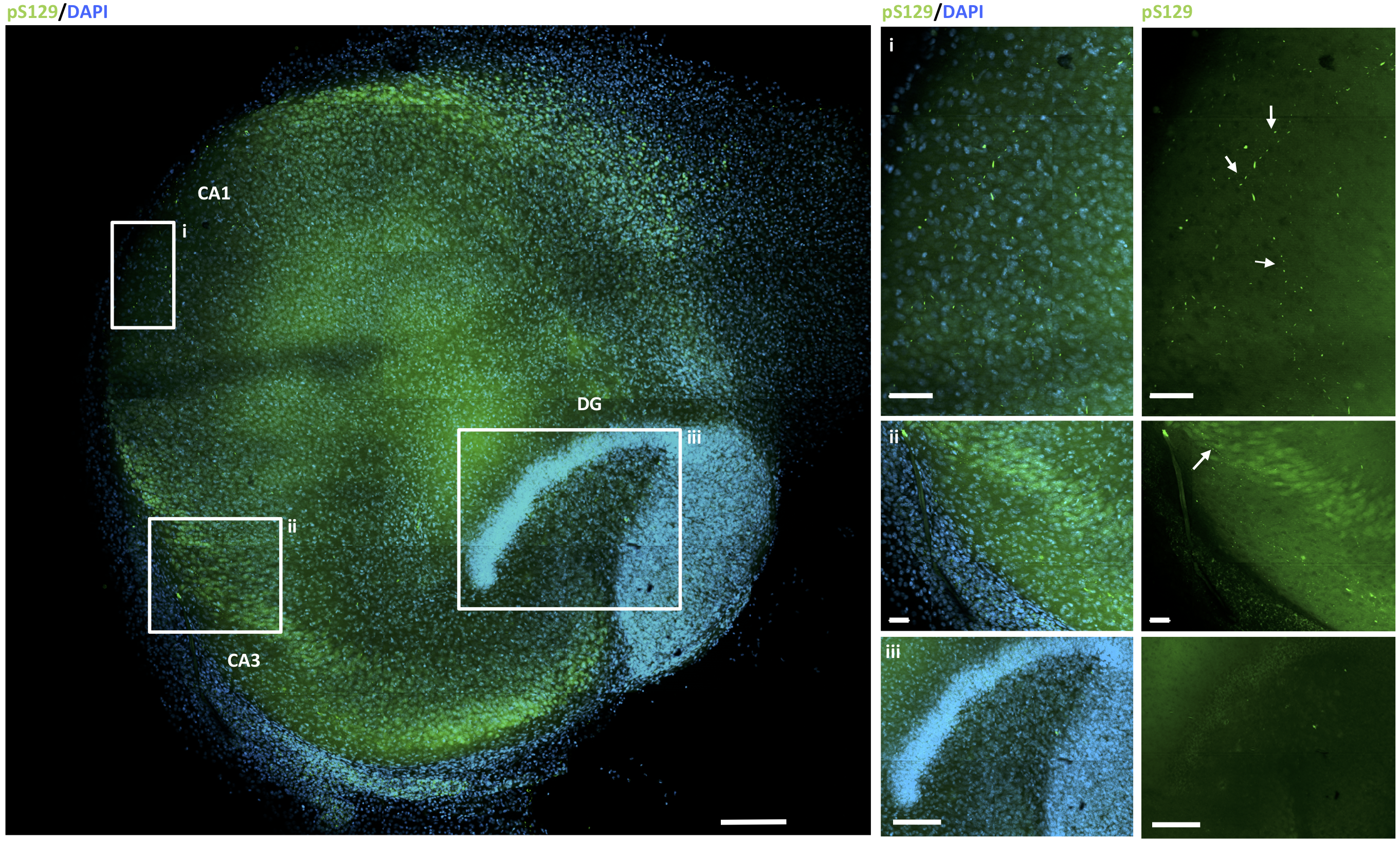

Supplement: Supplementary file 6 — Additional file 6: Figure S6. Composite image of WT OHSC with S129A PFFs applied as a drop on the surface of the slice 7 days post application, showing pS129-positive aggregates (D1R1R) found only at the periphery of the slice. Scale bar: 200 μm. Magnified images show the aggregates at the periphery of the slice in CA1 (i) and CA3 (ii). No aggregates were detected at DG region (iii). Scale bars for i & ii: 50 μm, iii: 200 μm. Images are representative of 3 experiments/14 slices in total. [file 40478_2019_865_MOESM6_ESM.tiff]

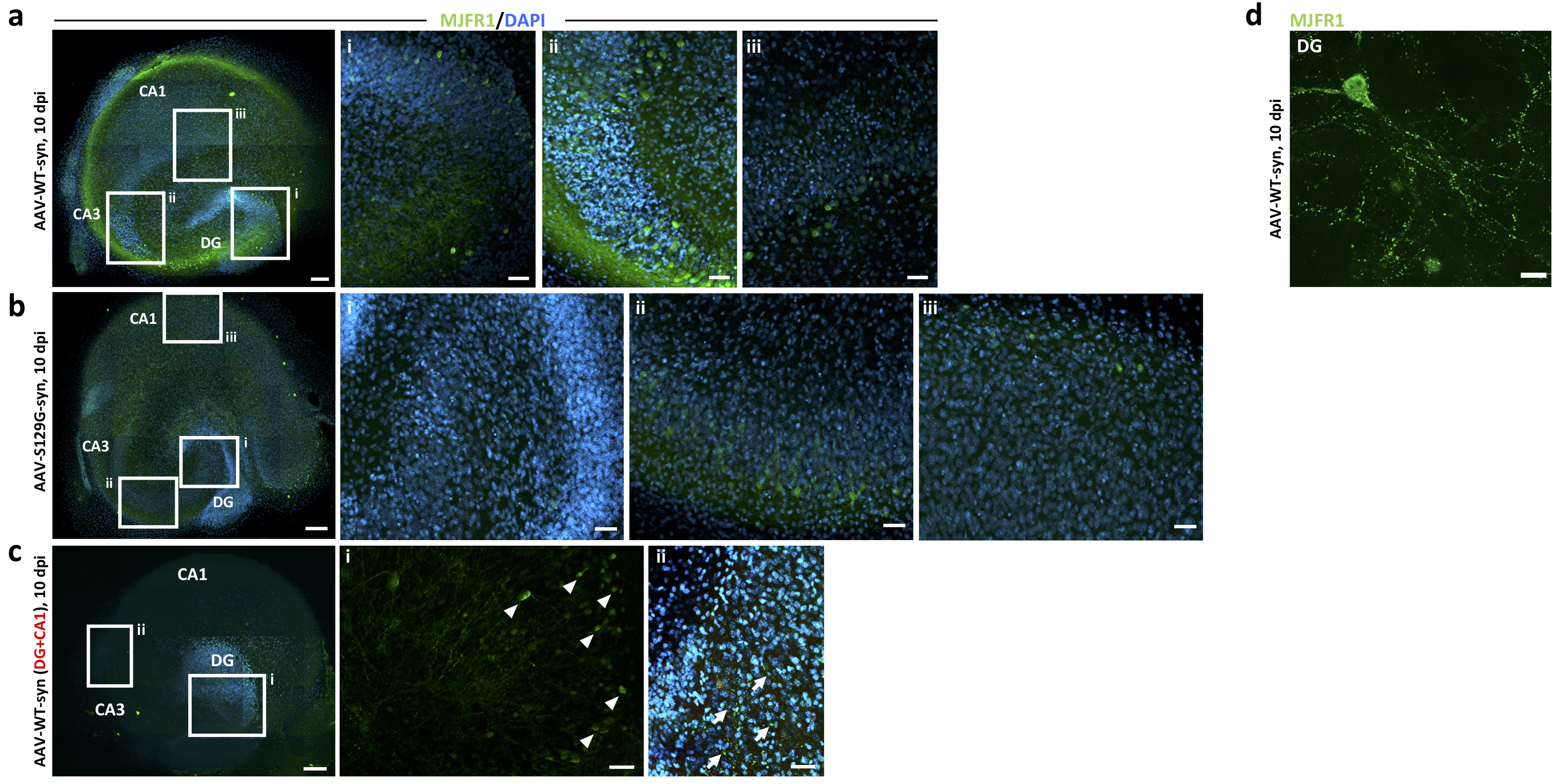

Supplement: Supplementary file 7 — Additional file 7: Figure S7. AAV-construct injection results in ample α-syn expression. a, b At 10 days post injection of AAV-α-syn (corresponding to 14 DIV), both the WT variant (a) and the S129G variant (b) give rise to a robust α-syn expression in all transfected regions, as detected by total human α-syn antibody MJFR1 (green). Panels show magnified images from the DG (i), CA3 (ii) and CA1 (iii), displaying α-syn positive neurons. Scale bars: 200 μm, insets: 50 μm. c Transfection with AAV in only the DG and CA1 results in α-syn expression limited to these areas (i). In the CA3 region, the proximal part displays a punctate α-syn staining resembling synaptic terminals (ii, arrows), while the distal part is clear of staining (ii). No cell body staining in the CA3 is seen (ii). Arrowheads designate α-syn positive cell bodies. Scale bar: 200 μm, insets: 50 μm. d High magnification image showing the α-syn distribution inside a transfected neuron in the DG. The punctate staining indicates an efficient sorting of the expressed α-syn. Scale bar: 20 μm. Images are representative from 3 to 5 individual experiments with 13–20 slices per group. [file 40478_2019_865_MOESM7_ESM.tiff]

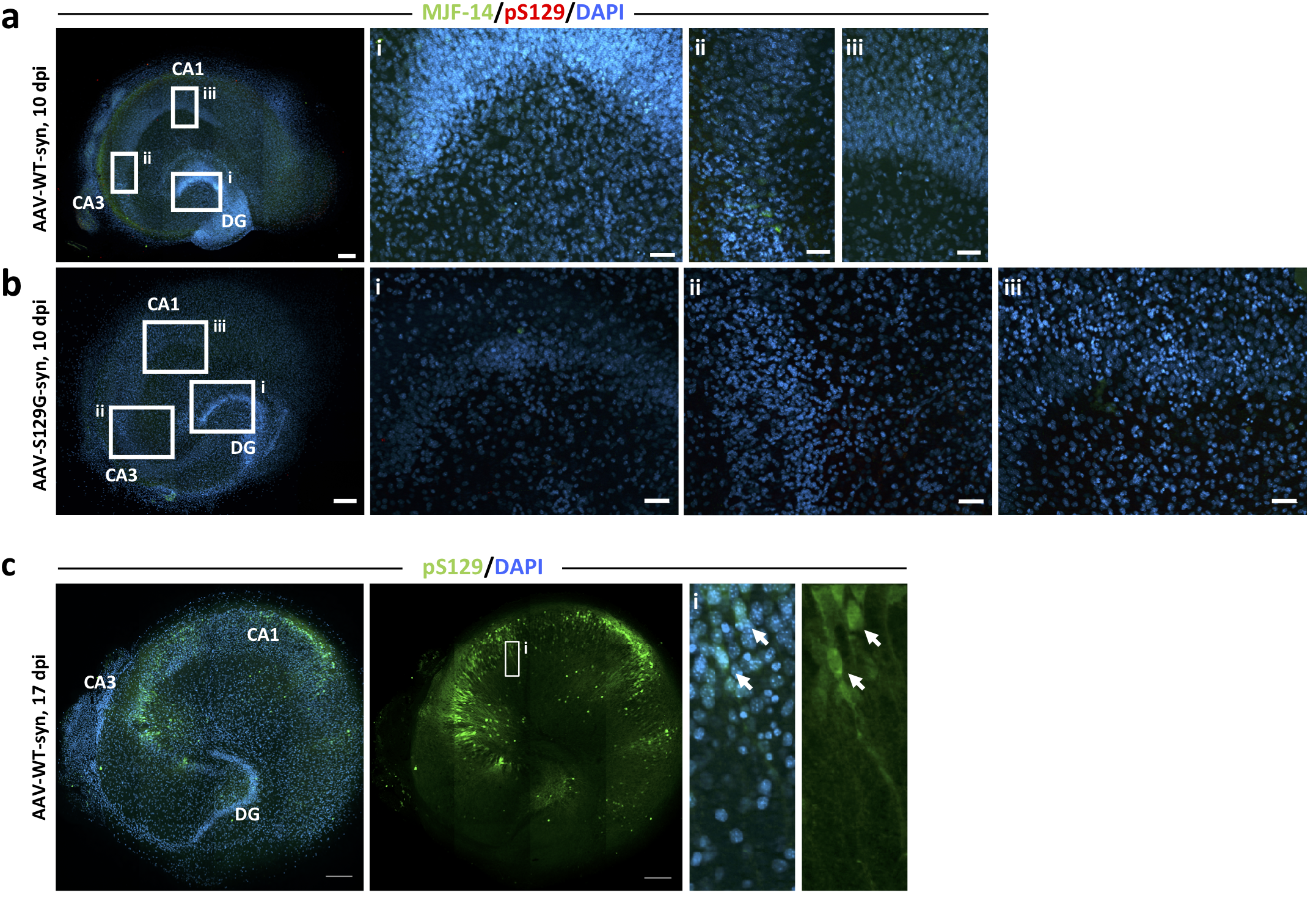

Supplement: Supplementary file 8 — Additional file 8: Figure S8. AAV-mediated overexpression of α-syn does not result in α-syn aggregation. a, b Staining for aggregated α-syn (MJF-14, green) and pS129-α-syn (11A5, red) at 10 days post transfection (14 DIV) does not detect any aggregation in either AAV-WT-α-syn (a) or AAV-S129G-α-syn slices (b). Insets show magnified images from DG (i), CA3 (ii) and CA1 (iii). Scale bars: 200 μm, insets: 50 μm. c At 17 days post transfection (21 DIV), a weak pS129-staining of particularly the pyramidal neurons of CA regions was seen in slices transfected with AAV-WT-α-syn. Magnified images show the co-localization of diffuse cell body pS129-staining with DAPI-stained nuclei. Scale bars: 200 μm, inset: 10 μm. Images are illustrative examples from 3 to 5 separate experiments/13–20 slices in total per group. [file 40478_2019_865_MOESM8_ESM.tiff]

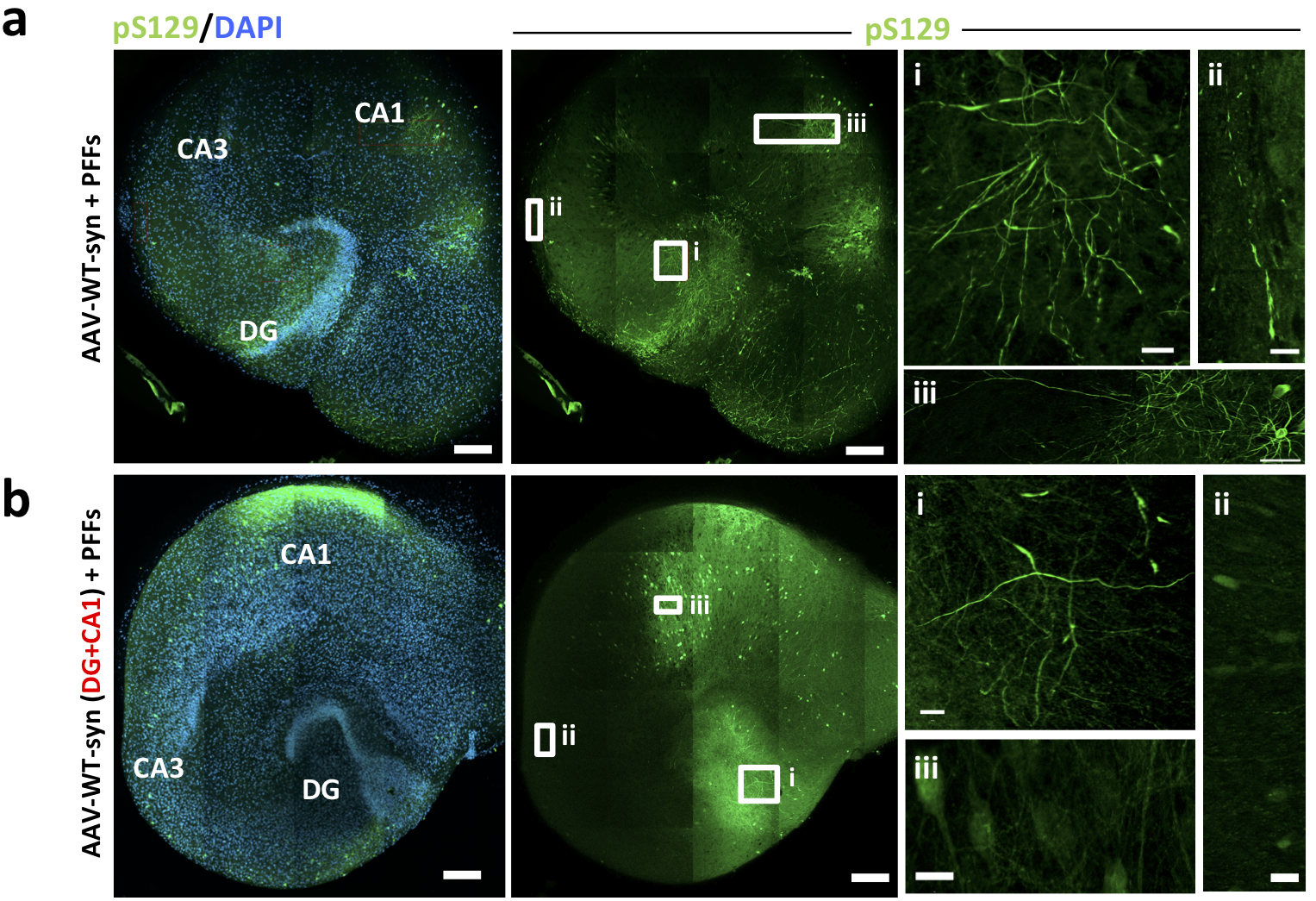

Supplement: Supplementary file 9 — Additional file 9: Figure S9. a 14 dpi of S129A PFFs in AAV-WT-α-syn expressing slices, robust aggregation and spreading throughout the hippocampal slice is detected by pS129-staining (D1R1R). Panels show magnified images of aggregates from DG (i), CA3 (ii) and CA1 (iii). Scale bars: 200 μm, i: 20 μm, ii & iii: 10 μm. b At the same time in slices only expressing AAV-WT-α-syn in the DG and CA1, leaving the CA3 blank of expression, aggregation in the DG is seen (i), equal to the slices expressing α-syn throughout the circuit. However, no aggregation is seen in either CA3 (ii) or CA1 regions (iii). Scale bars: 200 μm, i: 20 μm, ii & iii: 10 μm. Representative images from 3 independent experiments with 12–16 slices per group. [file 40478_2019_865_MOESM9_ESM.tiff]
